# Development and validation of a cynomolgus macaque grimace scale for acute pain assessment

**DOI:** 10.1038/s41598-023-30380-x

**Published:** 2023-02-24

**Authors:** Emilie A. Paterson, Carly I. O’Malley, Carly Moody, Susan Vogel, Simon Authier, Patricia V. Turner

**Affiliations:** 1 Department of Pathobiology, University of Guelph, Guelph, ON Canada; 2 Global Animal Welfare and Training, Charles River, Wilmington, MA USA; 3 Veterinary Services, Charles River Montreal, Senneville, QC Canada; 4 Veterinary-Safety Pharmacology Services, Charles River Laval, Laval, QC Canada; 5 Present Address: Department of Animal Science, University of California Davis, Davis, CA USA

**Keywords:** Behavioural methods, Preclinical research

## Abstract

Cynomolgus macaques may undergo surgical procedures for scientific and veterinary purposes. Recognition and assessment of pain using validated tools is a necessary first step for adequately managing pain in these primates. Grimace scales are one means of assessing the occurance of acute pain using action units such as facial expressions and posture. The aim of this study was to create and validate a Cynomolgus Macaque Grimace Scale (CMGS). Cynomolgus macaques (n = 43) were video recorded before and after a surgical procedure. Images were extracted from videos at timepoints at which breakthrough pain might be expected based on analgesic pharmacokinetics. Using the CMGS images were scored by 12 observers blinded to animal identification, times, and conditions. To validate the tool, detailed behavioral analyses emphasizing changes to baseline activity ethograms were compared to grimace scores. Four action units were identified related to potential pain including orbital tightening, brow lowering, cheek tightening, and hunched posture. The CMGS tool was found to have moderate inter- (ICC_average_ action unit mean ± SD: 0.67 ± 0.28) and good intra- (ICC_single_ mean ± SD: 0.79 ± 0.14) observer reliability. Grimace scores increased significantly (p < 0.0001) in the first four post-operative timepoints compared to baseline, correlating with behavioral findings (rho range = 0.22–0.35, p < 0.001). An analgesic intervention threshold was determined and should be considered when providing additional pain relief. The CMGS was shown to be a reliable and valid tool; however, more research is needed to confirm external validity. This tool will be highly valuable for refining analgesic protocols and acute peri-procedural care for cynomolgus macaques.

## Introduction

Nonhuman primates are often used in research and may undergo painful procedures as part of regular veterinary care and in scientific protocols. Research with nonhuman primates is highly regulated, requires ethical approval to conduct, and generally requires the use of analgesic drugs following painful procedures^1–4^. Veterinarians and scientists do the best they can to provide pain management for nonhuman primates in research settings with the information currently available. However, there are very few pharmacokinetic and efficacy studies demonstrating therapeutic analgesic blood drug levels in nonhuman primates, and none for which analgesic agents are combined, a technique used to provide greater analgesic coverage in other species^4–7^. Most analgesic recommendations for nonhuman primates are extrapolated from other species and it has been demonstrated that these extrapolations are not always accurate due to factors such as species-specific metabolism^7^. Further, analgesic protocols are often poorly reported, in part, due to the lack of pain assessment tools that would justify pain treatment^8^. Thus there are challenges in treating pain in nonhuman primates due to difficulties with interpretation of observations, a lack of validated pain assessment methods, unknown species-specific pharmacokinetic data, and no specific analgesia efficacy testing^7^. Cynomolgus macaques are widely worked with in research^9^, making development of a pain grimace score in this species highly impactful.

To provide effective pain management, it is essential that pain be recognized and assessed. Facial expressions have been used to evaluate acute pain in human medicine for verbal and non-verbal patients as well as in veterinary medicine using validated grimace scale tools^10–14^. Because of the similarity in aspects of manifestations of acute pain expression across species, it has been speculated that these changes have an evolutionary basis and are intended to elicit empathy, care and possibly communicate increased vigilance or alertness on behalf of an observer^15–18^. This latter point is emphasized by studies demonstrating a faster onset of facial grimacing following a painful procedure in mice exposed to conspecifics that have experienced acute pain as well as avoidance by study rats of experimental test areas showing pictures of faces or bodies of other rats in pain^19,20^. A grimace scale is typically composed of three or more action units—elements of the facial expression or posture that are reliably noted to change from baseline when a person or animal is in pain^18^. Examples include eye aperture opening, cheek muscle tightening and whisker position^14,18^. Ear position has also been a helpful action unit for some species with large, mobile ears that are visible on a frontal face view^19,21–23^, and neck tightness and/or head position relative to shoulders have also been described as relevant postural action units for humans, sows, and rhesus macaques^24–26^. The mouse grimace scale was the first grimace scale developed for animals^19^ and was followed by many others, including rats^21^, rabbits^27^, ferrets^28^, lambs^29^, sheep^30^, piglets^22,23^, horses^31^, and cats^32^ (see^33^, for a general review on the development and utilityof grimace scales for pain assessment in laboratory animals). Recent research is focused on refining and further validating these scales for real-time use for various types of clinical procedures or surgeries or for pain assessment^34^. For example, the mouse and rat grimace scales have been validated for cageside use as well as efficacy testing of analgesic protocols^35–37^. Through the use of these scales it was demonstrated that commonly used analgesic dose recommendations for rodents were insufficient, ultimately helping to improve pain management in these species^35–37^.

Grimace scales are typically developed using still images, which are then evaluated to ensure that they measure the construct of interest and have adequate validity and reliability. Six categories of measurements should be considered when developing and validating a novel pain assessment tool, and these include internal consistency, reliability, measurement error, criterion and construct validity, and responsiveness^14,18,38^. Construct validity must be assessed to ensure that the tool is measuring pain. To do so, grimace scores are compared to validated measures of pain. For example, during development of the feline grimace scale, grimace scores obtained in presumed painful cats were compared to pain scores obtained with the ‘Glasgow composite measure acute pain scale-feline’, a validated pain assessment instrument in cats^32^. Primates do not have a validated pain assessment tool and behavior is frequently used for pain assessment, for example, evaluating decreased activity or guarding of limbs in painful animals^7,26,39,40^. However, assessing behavior can be labor intensive, is challenging to conduct with a large group of animals in a research setting, and requires specific training and oversight to do well. Another downside is that human presence can alter behavior in animals, emphasizing the importance of assessing behavior indirectly^41^. The reliability of each grimace scale must be examined to ensure that a tool can produce similar repeated measures over time and when used by different individuals. To do so, multiple individuals blinded to the experiment score the same set of images to assess if each individual can produce similarly reliable results^42^. Values related to validity and reliability are essential; however, they do not always translate into clinical significance. It is preferred to develop or refine grimace scales in a clinical setting and use opportunistic sampling (i.e., animals involved in ongoing studies/procedures) so that the research is readily translatable, has clinical relevance as well as contributes to reduction of numbers of animals worked with, an important ethical consideration. Finally, to improve the clinical applicability of the tool and address measurement error it is useful to identify an analgesic threshold in which the grimace score can used to identify when potential pain should be treated^34,42^.

As mentioned, there are currently no validated pain assessment tools for nonhuman primates, although there has been recent work defining general animal welfare indicators for nonhuman primates held in biomedical research environments^43,44^. Most of the indicators identified are related to animal-based and environment-based measures that are not specific to pain. Descovich and others examined wellness indicators and post-operative pain in rhesus macaques and identified possible facial action units related to pain, including orbital and lip tightening. However, the overall results of their study were not significant, possibly because of small group sizes and a range of procedures being evaluated^26^. This work, although preliminary, emphasized the importance of using facial expressions together with behavior and postural changes when evaluating macaques for possible pain^26^.

Even when human or animal patients are treated with analgesics, there is a moderate probability that breakthrough pain will occur^26,43^. Breakthrough pain has been reported in human and animal patients following surgery despite the use of analgesics from 1 to 8 h and up to 24 h post-surgery for a number of reasons, including imperfect tools for assessing and treating analgesia needs^45–47^. It would be unethical to conduct surgery on primates to develop a pain assessment tool without providing appropriate analgesia relief. However, timepoints can be estimated for when breakthrough pain might occur, for example, just prior to scheduled readministration of analgesia when blood drug levels are waning. The timing for these assessments can be based on the pharmacokinetics of the analgesic agents being administered[see for example ^7,26,40^. This approach was used in a recent study that attempted to correlate pain-associated behaviors (e.g., slumped posture, decreased activity) with facial expressions (e.g., orbital tightening) in rhesus macaques that had undergone painful procedures before subsequent doses of analgesics were administered^26^.

This study was conducted in two parts, first a proof-of-concept phase followed by a validation phase, and aimed to develop a Cynomolgus Macaque Grimace Scale (CMGS) to detect acute or breakthrough pain in the post-operative period. More specifically the objectives were to (1) determine facial and postural action units related to post-operative pain in the cynomolgus macaque to create a CMGS; (2) examine the construct validity, internal consistency, and the reliability of the CMGS; (3) to assess the criterion validity of the CMGS by comparing with detailed behavioral analyses during the baseline and post-operative periods, and 4) apply the CMGS to develop a threshold score at which additional analgesia administration should be considered. We hypothesized that (1) when breakthrough pain is probable, specific facial or postural action units will be present that can be used to differentiate from the individual’s baseline state; (2) when primates experience breakthrough pain in the post-operative period, CMGS scores will be higher compared to baseline and that the scores from the same observer (intra-observer reliability) and different observers (inter-observer reliability) will be similar; and (3) there will be an increase in pain-associated behaviors and a decrease in overall activity as well as elevated CMGS scores in the post-operative period when compared to baseline, demonstrating good criterion validity.

## Methods and materials

### Animals

All animal use and procedures were reviewed and approved by the Charles River Senneville (CR-SEN) Institutional Animal Care and Use Committee (protocol #30768) and conducted at the Charles River Laval facility (Laval, QC, Canada). The pre-clinical facilities are accredited by the Canadian Council on Animal Care (CCAC) and AAALAC International, and procedures and practices were performed in accordance with the CCAC Guidelines and followed Good Laboratory Practices (GLP). The animal study complied with the ARRIVE Guidelines^48^. Opportunistic sampling was employed as animals were undergoing a planned surgery (telemetry transmitter instrumentation for electroencephalograph and electromyograph monitoring) for subsequent sponsored GLP studies. Inclusion criteria were as follows: captive-bred cynomolgus macaques of a consistent age and weight by sex, undergoing the same EEG and EMG telemetry instrumentation procedure (a moderately invasive surgical procedure), and receiving a multimodal analgesic regimen. Group size was established a priori based on randomization, number of primates and cages available, and social compatibility.

Healthy captive-bred juvenile cynomolgus macaques (*Macaca fascicularis*) (N = 43, 22 M and 21F), average age 2.2y (2.1–2.2y) and average body weight of 2.0 kg (1.7–2.4 kg) were enrolled (see supplementary material, Table S1). A within-subject study design was employed and animals acted as their own controls. Macaques were randomized and socially housed in pairs (n = 14), trios (n = 4), or quartets (n = 1) during the baseline and post-operative periods except for one male who was separated approximately 12 h post-surgery due to behavioral issues and subsequently socially re-housed (see supplementary material, Table S1). Macaques were housed in stainless steel cages with four quadrants in which pairs had access to two horizontal quadrants and trios and quartets had access to all four units. Each quadrant had an internal surface of 0.38 m^2^; internal volume of 0.31 m^3^; height of 0.83 m (Allentown Cages, Allentown, NJ, USA). Pairs had access to two metal perches (i.e., a platform) approximately 20 cm above the cage floor and trio/quartets had access to four perches in which three were 20 cm above the cage floor and one was 1 m above the cage floor. Each cage was outfitted with a stainless steel swing and contained at least one manipulable item per animal (Bio-Serv, Flemington, NJ, USA).

The room environment was set to maintain a temperature of 21 ± 3 °C and relative humidity of 50 ± 20%, with a 12 h/12 h light:dark cycle (lights on: cohort 1: 07:00, cohort 2: 06:30). UV-treated, reverse osmosis water was available ad libitum from multiple lixits per cage. Standard certified chow (Envigo Teklad Certified Hi-Fiber Primate Diet #7195C, Indianapolis, IN, USA) was provided twice daily. Certified treats (Bio-Serv, Flemington, NJ, USA) and fresh or frozen fruits were given daily in a variety of puzzle feeders and foraging devices. Nutritional support was provided at least three days prior to surgery and for at least one week post-surgery consisting of bananas and certified chow mixed together, a daily bowl of three different fruits/vegetables, and a multivitamin (Flintstones Vitamins, Bayter Inc, Mississauga, ON, Canada).

### Surgical procedures and perioperative care

Surgical procedures for telemetry instrumentation were identical to those described in^49^. Briefly, a sterile transmitter was inserted subcutaneously and from this transmitter electroencephalograph (EEG) leads are tunneled subcutaneously to the back of the neck. A linear groove was made on the cranial cortical bone to secure the electrodes onto the external surface of the cranium. Electromyelograph (EMG) electrodes were positioned and secured parallel to the longitudinal axis of the neck muscle. Surgery for cohort 1 occurred in October 2020 and for cohort 2 occurred in January 2021. The perioperative analgesia program differed slightly between cohort 1 and cohort 2 (Table 1). Animals were monitored during surgery every 10 min until full recovery and returned to their home cage approximately 1 h after surgery end, ensuring that body temperature was > 37.0 °C.

**Table 1:** Multimodal perioperative analgesic regimen for cohort 1 and 2 animals.

Cohort 1	Drug	Dosage (Conc.)	Frequency	Route
Pre-operative	Ketamine + acepromazine	10 mg/kg + 1 mg/kg	1	IM
	Buprenorphine	0.06 mg/animal	1	IM
Perioperative	50:50 Bupivicaine and lidocaine	max of 0.1 mL/site (0.25%: 20 mg/mL)	1	SC
Post-operative	Buprenorphine	0.06 mg/animal	Every 12 h for 3 days	IM
	Meloxicam	0.2 mg/kg	1	SC

Cohort 2	Drug	Dosage (Conc.)	Frequency	Route
Pre-operative	Ketamine + acepromazine	10 mg/kg + 1 mg/kg	1	IM
	Meloxicam	0.2 mg/kg	1	SC
	Buprenorphine	0.02 mg/kg	1	IM
Perioperative	Bupivacaine	0.25%,0.5 mL (max of 1.2 mL/site, up 2.4 mL)	1	SC
	Buprenorphine-sr	0.2 mg/kg	1	SC
Post-operative	Meloxicam	0.1 mg/kg	Every 22–24.5 h (min 3 doses)	PO

### Proof-of-concept and validation of the CMGS

#### Behavior recording and scoring

**Figure 1:**
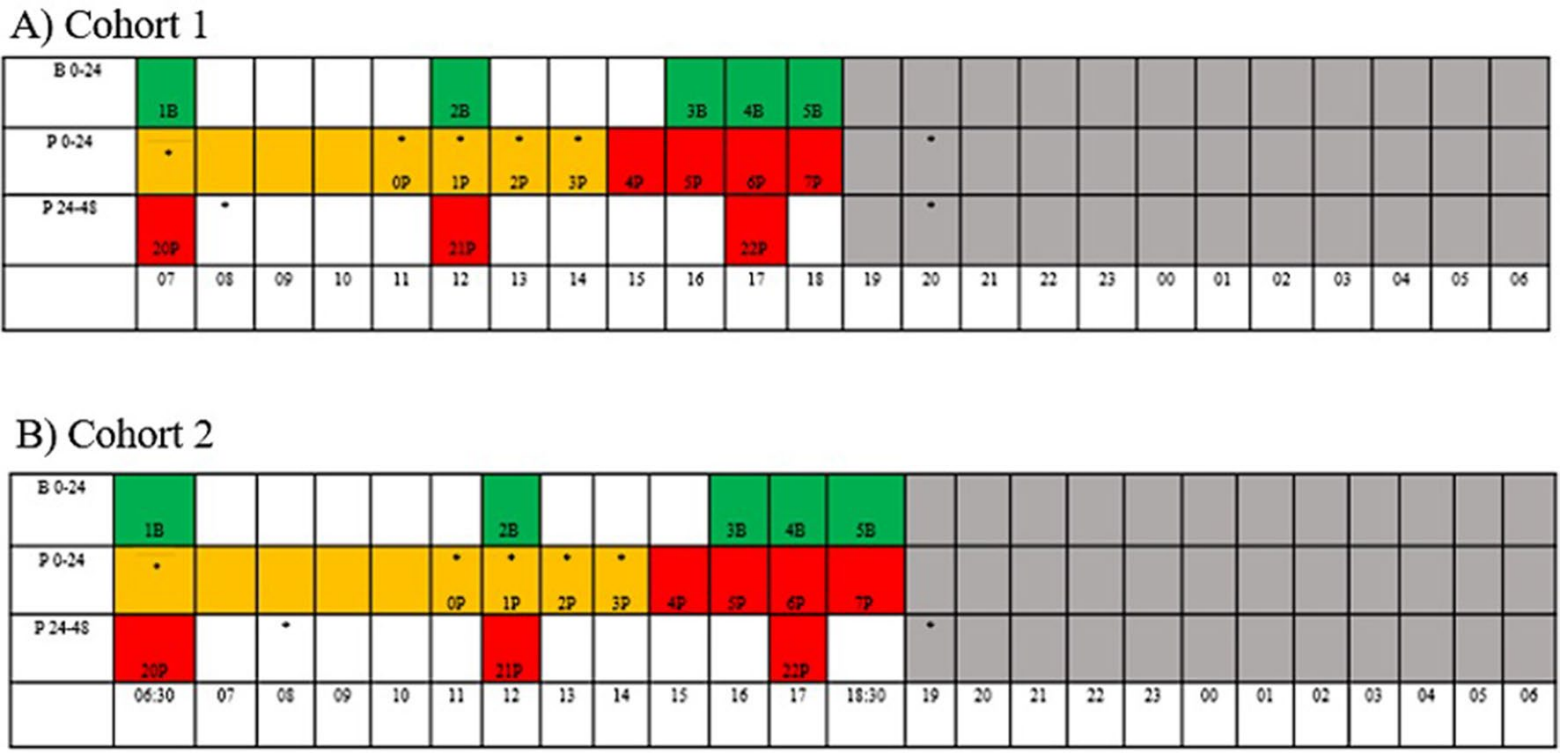
Overview of peri-operative observation and treatment of cynomolgus macaques. Twenty-four hour baseline (B 0–24) and 48 h post-surgery (P 0–24, P 24–48) video recording capture representation of cohort 1 (**A**) and cohort 2 (**B**). Cohort 1 day light cycle: 07:00 to 19:00; cohort 2 day light cycle: 06:30 to 18:30, dark phase indicated by grey squares. Every square represents an hour (unless indicated otherwise). Green squares indicate the 15 min period of the hour that was observed and scored during baseline, red squares indicate the 15 min period of the hour that was observed and scored during the post-operative period, and yellow squares indicate the surgical period. The approximate time of analgesic dosing is indicated by a (*) and varied per individual during the surgical period.

Individuals were recorded for 24 h in their home environment (number of cages = 19) up to one week prior to surgery (control) and then immediately after surgery for 48 h using high-definition surveillance cameras (5 megapixels PoE Fixed Bullet RLC-410-5MP, Reolink Digital Technology Co., Ltd., China) mounted on a custom stainless steel and plexi-glass frame that attached to each quad unit of a home cage (see supplementary material Table S1 for subject characteristics and housing groups and Fig. 1 for timepoints of observations and treatments). Video footage was recorded at a rate of 20 frames per second (FPS) and at a resolution of 2048 × 1536. When video recordings took place, people were not in the room and the recordings were stopped during technical and husbandry activities. Video footage was used for image collection, sampling strategy, and behavioral observations. Prior to recording baseline behavior, the custom mount was attached to the cage doors for a 24 h habituation period. Behavior was scored using continuous focal animal sampling of all of behavior patterns listed in the ethogram (Table 2, adapted from^20^) for the first 15 min/hour by an observer (EAP) blinded to primate identification, day, and time using Observer XT (version 15.0.1200: Noldus Information Technology, Wageningen, the Netherlands). Note that because primate hair was clipped for surgery the observer could not be completely blinded to condition (i.e., pre- vs post-clip). However, scorers were not aware that clippings indicated that primates were in a post-op condition (i.e., during training clipping was not discussed, and at this facility, animals may be clipped up to two days prior to surgery). Video recordings were randomized (random.org) and a total of 111 h of continuous focal data was scored.

**Table 2:** Primate behavior ethogram (adapted from^19^).

Category	Behavior	Descriptions	Duration or Frequency
Locomotion	Climbing	Climb vertically or descent vertically, hanging from cage top or cage enrichment and limbs are not touching the cage floor or main perch	Duration
	Inactive	The primate is sitting without movement or appears to be sleeping	Duration
Self-maintenance	Self-groom	The primate is manipulating fur with hands or mouth, displacing the fur, picking, plucking, licking, scratching, or thoroughly examining skin or fur	Duration
	Forage	The primate searches for food	Duration
	Eat	The primate consumes food	Duration
	Drink	The primate consumes water from lixit or cage floor	Duration
Resource- directed	Manipulate cage resources	The primate uses hands, feet or mouth to manipulate or carry resources provided in or on the exterior of the cage	Duration
Affiliative	Allo-grooming	The animal manipulates a cage mates’ fur with hands or mouth, displacing the fur, picking, plucking, licking, or thoroughly examining skin or fur	Duration
	Play	Animals will pull, poke or mock bite a cage mate	Duration
	Embrace/huddle	Ventral-ventral, dorsal–ventral, or distal/proximal surface of body touching or holding of companion	Duration
Agonistic	Aggression	Primate performs aggressive behavior towards a cage mate (i.e., displacement, chase, mount, threat, bite)	Duration
Abnormal	Abnormal repetitive behavior	The primate performs abnormal repetitive behavior which may be a locomotor stereotypy, appetitive stereotypy, self-directed or self-injurious	Duration
Sedation	Rub face	Primate rubs a part of its face using hands or feet	Duration
	Tremor	Rhythmic movements of shivering or quivering	Duration
	Ataxia	The primate is stumbling, falling, and has overall uncoordinated movement	Duration
Pain	Movement directed towards the wound	Primate directs its behavior towards the surgical site our area of the body that was implicated in a procedure (i.e., scratching, licking, touching)	Duration
	Bruxism	Teeth grinding or jaw clenching can be observed by a chewing motion	Duration
	Body shake	Shaking up or down the body as if to remove particles or water	Frequency
	Hunched	Sitting with back curved, shoulders slumped, head may be lower than shoulders, chest may rest on knees and head may rest on cage bars (out of a social resting context)	Duration
	Head lean	The primate rests its head on a cage or cage furniture surface	Duration

#### Sampling strategy

The sampling strategy was determined by scoring 3 h of behavior at three different timepoints (two baseline and one post-operative) for three different cages and six macaques at sampling durations of 5, 10, 15 (starting at the top of the hour) and 60 min (see supplementary material, Table S6 for subject characteristics and time points). Behavior scoring consisted of continuously recording all behaviors. The percentage of time engaged in each behavior for the first 5, 10, and 15 min were compared to behaviors expressed over the entire 60 min period using a Spearman’s rank correlation^50^. It was determined that scoring for the first 15 min of each hour provided a reasonable compromise, matching 74% of behaviors occurring in any given hour (rho = 0.74, p < 0.05)^30^.

#### Image capture and grimace scale development

**Figure 2:**
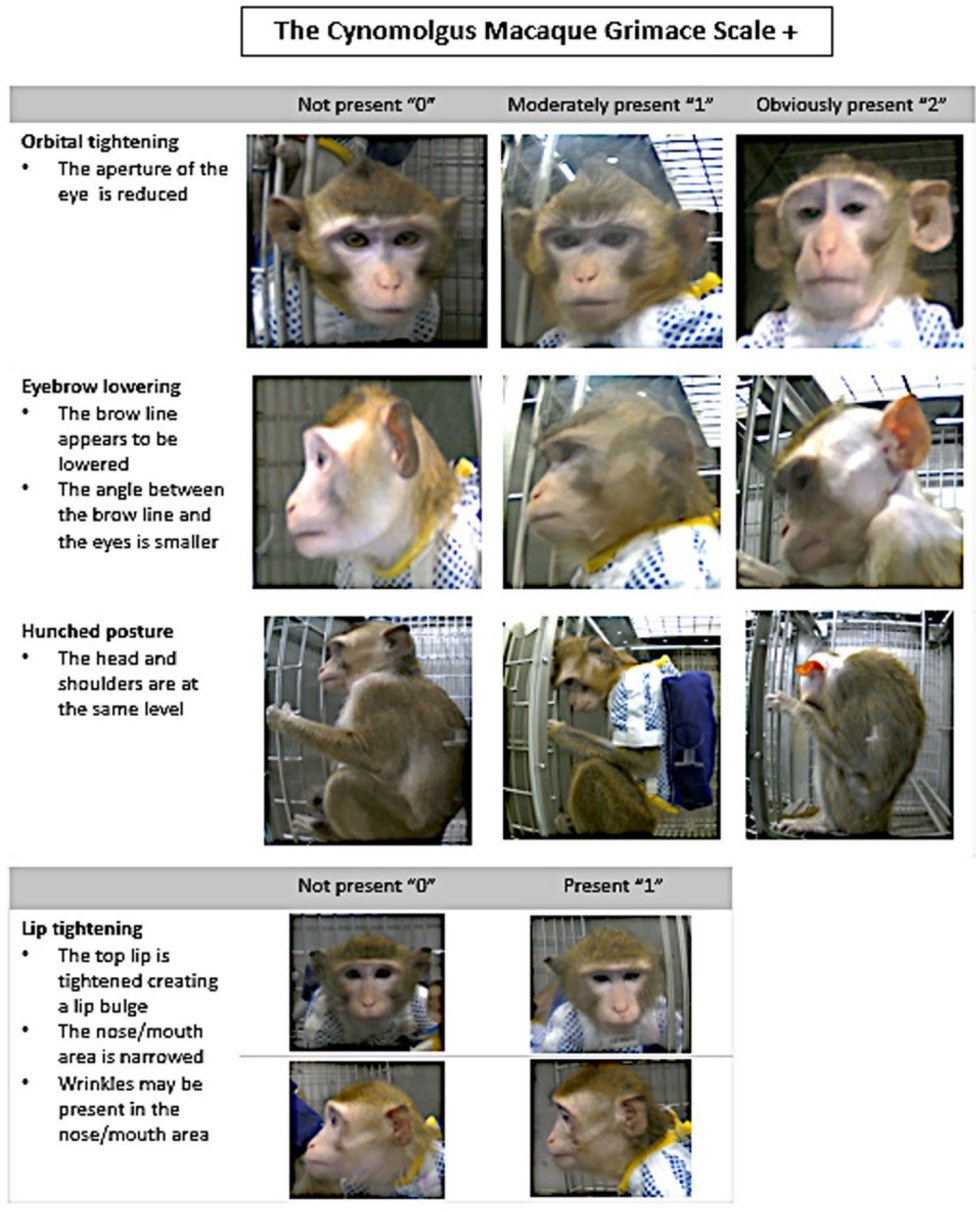
Cynomolgus Macaque Grimace Scores are significantly increased from baseline after surgery. Boxplot demonstrating median (solid line) and the interquartile range of the CMGS scores across timepoints pre- and post-surgery. The whiskers indicate the range and circles the extreme values. The lines in the x axis indicate that timepoints are not linear. The CMGS scores increased during the first four timepoints following surgery (pairwise comparison with Tukey’s adjustment, p < 0.0001). Timepoints start from up to 168 h before surgery (1B: 06:30–7:30 (cohort 1) or 07:00–08:00 (cohort 2), 2B:16:00–17:00, 3B:17:00–18:00, 4B:18:00–19:00) and up to 36 h post-surgery ((1P:16:00–17:00 (3 h post-op), 2P:17:00–18:00 (4 h post-op), 3P: 18:00–19:00 (5 h post-op), 4P: 06:30–07:30 (cohort 1) or 07:00–8:00 (cohort 2)(17 h post-op), 5P: 17:00–18:00 (24 h post-op) and 6P: 1B: 06:30–07:30 (cohort 1) or 07:00–08:00 (cohort 2) (36 h post-op)).

To create the CGMS, images of faces and bodies were compared at different timepoints post-surgery by two researchers (EAP, PVT) at times during which breakthrough pain might occur, based on published analgesic pharmacokinetics^20,23,28^ to the images taken from a given animal’s baseline video recordings, during which no pain was expected to be present[see ^18^ for a description of a similar process for development of the MGS]. Features that were consistently different were marked as facial action units, including brow lowering, and orbital and lip tightening as well as hunched posture. These action units were later described and developed into a training manual (see supplementary material Figure S2) to create the CMGS (Fig. 2). Brow lowering, orbital tightening, and hunched posture were defined using a 3-point score (0–2) and lip tightening was defined as a 2-point score (0–1). The different scoring scale was used because the action unit “lip tightening” could not be further subdivided reliably between ‘present’ or ‘absent’. The maximum CGMS score is seven and overall score values were transformed into a proportion for interpretation.

One person (EAP) unblinded to animal treatment (i.e., pre- and post-operative) selected frontal, profile, and whole-body images from video recordings (the same videos as the behavior observations) with screenshots collected only when the animal was in profile or directly facing the camera (see supplementary material, Tables S2 to S5 for image capture details per subject). Images were selected each hour between ~ 16:00–19:00 following surgery and on the following morning during the first hour of light, that evening from 17:00 to 18:00 (~ 24 h post-operative) and the following morning during the first hour of light (~ 36 h post-operative) (Fig. 1). Images were excluded if they fell within a 2 h window of returning to home cage post-surgery to avoid the potential for residual sedation. Images were retrieved from baseline video recordings (the same videos as the behavior observations) at four timepoints that were timed matched with the six post-op time points (two of which were repeated on subsequent days). Frontal and profile images were cropped to include the face only and edited for brightness and sharpness. In total, 1,940 images were collected (see supplementary material, Figure S1 and Tables S2–5). The number of images per subject varied because of differences in animal activity and positioning. For example, using time point 1P (from 16:00 to 17:00) for animal 1 M, EAP watched the video in real-time speed and paused the video recording everytime the primate looked directly into the camera, was positioned in a profile view or the full body could be observed with the goal of capturing as many pictures as possible during the chosen hour. Occasionally primates did not look at the camera for the duration of the video thus resulting in no facial images for that time point. To limit the potential for selection bias, all images from a given timepoint were used for scoring. Because facial expressions can be transient, an average grimace score over a given period is considered more representative of the overall state (i.e., more vs less pain). All video recordings were collected as scheduled; however, room maintenance or other study-related activities (e.g., body weights, detailed examination) for which primates needed to be temporarily removed from their cages resulted in 7 h of unusable footage.

#### Scoring images using the CGMS

Twelve volunteer employees (5 M, 7F) with significant primate technical experience (2–30 y, average 11 y) attended a 45 min virtual training session and were provided with the CMGS and training manual. Images were randomized (random.org), observers were blinded to animals (no knowledge of what animals’ numbers were and have not worked or seen these primates in person), times (no knowledge of timelaspe after surgery or the administration of analgesics), and condition (whether the primate was in the pre or post operative condition). Volunteers were split into 3 cohorts and each cohort was composed of 4 individuals. Each cohort received the randomized pictures with several repeated images to assess intra-rater reliability each week (see Figure S1 in the supplementary material). If the participants judged that they could not score a facial action unit or posture due to image quality or positioning they were instructed to indicate ‘not possible to score’. After scoring, participants completed an anonymous survey pertaining to their experience and the process of training and image scoring (see supplementary material, Survey questions; note that according to article 2.5 of the Canadian Tri-Council Panel on Research Ethics [https://ethics.gc.ca/eng/tcps2-eptc2_2018_chapter2-chapitre2.html#a] quality assurance and improvement information for training material that is collected anonymously and analyzed in aggregate is exempt from REB review).

#### Analgesic threshold determination

An analgesic intervention threshold is used to discriminate CMGS scores (from images of macaques) that would, in retrospect, signal a need for additional analgesic treatment^18,51^. The analgesic intervention threshold was used conjunction with the prescribed analgesic treatment and examined using a scatter plot. Three experienced primate veterinarians unfamiliar with the CMGS and blinded to timepoint, independently scored 295 randomized images (profile, frontal and whole body) as ‘pain’, ‘no pain’ or ‘not possible to score’. Images included seven timepoints (one baseline, six post-op) of every animal (n = 43). Timepoints 1B (pre-op), 5P (24 h post-op), and 6P (36 h post-op) were classified as not painful given that behavior changes were not significantly different from baseline. Timepoints 1P (3 h post-op), 2P (4 h post-op), 3P (5 h post-op), 4P (17 h post-op) were classified as painful since most behavior categories significantly differed from baseline.

### Statistical analyses

R studio (R core team, R Foundation for Statistical Computing, Boston, MA, USA) was used for all analyses. A threshold of p < 0.05 was used as the criteria for statistical significance.

The reliability of CMGS scores was assessed using the interclass correlation coefficient. The reliability of each action unit for every image was assessed, calculated for each cohort independently (comparing four observers) using a two-way random effects model for absolute agreement, based on a single and average measure with a 95% confidence interval. If two or more individuals chose the option ‘not possible to score’ for an action unit, this action unit was not evaluated. The intra-observer reliability was assessed by repeating three images/week (nine total) and analyzing the individual action unit using a two-way mixed effects model for absolute agreement, based on a single measure with a 95% confidence interval. Both ICCs were interpreted as follows; a score < 0.5 = poor reliability, 0.5–0.75 = moderate reliability, 0.75–0.9 = good reliability, and > 0.9 = excellent reliability^50,52^. A nonparametric test (Mann Whitney U test) with ICC as the numeric value and gender as the binary factor was used to evaluate gender bias. The rationale for examining this is evidence of gender bias in attitudes towards animals and concern about animal welfare, with female observers having more positive attitudes towards animals and more concern for welfare than male observers^53^.

Cronbach’s alpha coefficient was used to evaluate internal consistency and calculated for the final CMGS as well as each action unit based on the average score of all observers, recalculating the coefficient by removing each action unit. The interpretation was as follows: alpha value < 0.65 = unsatisfactory, 0.65–0.69 = fair, 0.7–0.74 = moderate, 0.75–0.79 = good, and > 0.8 = excellent^50^.

To assess the difference between CMGS at different timepoints (CMGS changes through time; construct validity) a Gaussian linear mixed model and pairwise comparison were conducted. CMGS scores were composed of the average of all action units scored for a given animal at a specific timepoint. CMGS were assessed for normality using a Q-Q plot. An ANOVA was used to examine the effects of timepoint per condition, sex and cohort on CMGS scores. A significant effect of timepoint, sex and cohort were found on CMGS scores and used to calculate the least-square means (LSM). An interaction between sex and timepoint per condition as a fixed effect was tested with no significant effect on CMGS scores and was removed. A Gaussian linear mixed model with timepoint per condition, sex, and cohort as fixed effects and a random effect of subject nested within cage was used to calculate LSM. The random effect of subject nested within cage takes into consideration the repeated measures of the individual subject housed in a cage in which cage is the experimental unit. Pre- and post-op CMGS scores were compared with a pairwise comparison with timepoint per condition, sex and cohorts as fixed effects and subject nested within cage as the random effect. P values were corrected for multiple comparison using a Tukey’s adjustment.

To determine criterion validity, descriptive analysis of all behaviors examined was performed to exclude low frequency behaviors (i.e., drink, aggression, abnormal repetitive behavior, rub face, tremor, bruxism, and head lean). Normality was assessed by visually examining Q-Q plots and all behaviors that did not meet the assumption of the linear mixed model (homogeneity of variance) were square-root transformed except for proportion of time spent active/inactive, which was distributed normally. Behavior groups were the response variables in the model described below. The interaction between sex and timepoint was tested as a fixed effect and did have an effect on some behaviors and was kept in the final model. A Gaussian linear mixed model with timepoint per condition, sex, interaction between sex and timepoint per condition, and cohort as fixed effects and a random effect of subject nested within cage was used to determine the LSM and to compare pre- and post-operative measures using a pairwise comparison with a Tukey’s adjustment. The within subject design or repeated measures of an animal at different timepoints is controlled statistically using the random effect of subject nested within cage in which cage is the experimental unit. The proportion of duration (time a specific behavior is observed/time of the video recording observed) of behaviors that were assessed with the Gaussian linear mixed model were grouped into four categories and summed (values may exceed 1); positive species-typical behavior includes forage, play, and manipulation of cage resources; general activity/maintenance includes active/inactive and eat; social behavior includes embrace/huddle and allogrooming, and pain-associated behavior includes movement directed towards the wound, hunched, and self-groom. After identifying which behaviors were not expressed by the cynomolgus macaques in this study, the rationale for grouping behaviors was based on expert (EAP, PVT) opinion and the literature^26^. Behaviors in each group were related (i.e., interaction with conspecific categorized as social behavior) and statistically moved together (i.e., correlated) so that there was no counteracting effect. A Pearson correlation was used to assess the relationship between CMGS scores and the four categories of behavior (rho and p values).

To determine the analgesic threshold, a receiver operating characteristics (ROC) curve analysis was conducted. An area under the curve (AUC) of 0.5 was used to compare the area under the ROC curve^51^. Specificity and sensitivity values are presented with 95% confidence intervals. The ROC curve was generated by plotting the true positives (sensitivity) on the y axis and false positives (1-specificity) on the x axis. The ‘true state’ was determined based on behavior scoring. If a primate image was classified as ‘pain’ when the image corresponded to a timepoint classified as ‘pain’, a true positive was obtained. If a primate image was classified as ‘pain’ when the image corresponded to a timepoint classified as ‘no pain’, a false positive was generated.

## Results

### Experiment 1: CMGS—proof-of-concept

All animals demonstrated stable physiologic parameters during surgery. One female macaque died in the post-operative period while under anesthesia for unknown reasons and was excluded from this study. The surviving cagemate was paired with a naive female (behavior was not scored for the naïve animal). Anesthetic duration (from induction to the stop time of isoflurane) for cohort 1 was 192 ± 3 min and for cohort 2 was 159 ± 2 min. Surgery duration (from the first incision to the closure of the last incision) for cohort 1 was 121 ± 1 min and for cohort 2 was 84 ± 1 min. Extubation to re-establishment of physiologic parameters duration (finish time of surgery to the time back to homecage and start of video recording) for cohort 1 was 79 ± 1 min and for cohort 2 was 71 ± 1 min.

#### CMGS scoring reliability

Observers collectively scored 1,940 images from 10 timepoints (four baseline, six post-op). The inter-observer reliability of the CMGS scores varied across action units demonstrating that some action units were more reliable than others (Table 3). ICC_average_ scores demonstrated higher reliability than the ICC_single_ for each action unit and the overall CMGS scores suggesting increased reliability when multiple observers assess the same animal. Lip tightening demonstrated poor reliability (ICC_average_ range: 0.09–0.46), brow lowering demonstrated moderate reliability (ICC_average_ range: 0.54–0.78), orbital tightening demonstrated good reliability (ICC_average_ range: 0.78–0.84), and hunched posture demonstrated excellent reliability (ICC_average_ range: 0.92–0.93).

**Table 3:** Inter-observer reliability of CMGS scores across three cohorts from 12 raters.

Action unit			Cohort 1 ICC (95% CI)	Cohort 2 ICC (95% CI)	Cohort 3 ICC (95% CI)
Orbital tightening	Week 1	ICC_single_	0.57 (0.45–0.67)	0.46 (0.34–0.58)	0.63 (0.52–0.72)
		ICC_average_	0.84 (0.76–0.89)	0.78 (0.66–0.85)	0.87 (0.81–0.91)
	Week 2	ICC_single_	0.43 (0.29–0.56)	0.42 (0.24–0.57)	0.56 (0.35–0.70)
		ICC_average_	0.75 (0.61–0.84)	0.74 (0.52–0.85)	0.83 (0.66–0.91)
	Week 3	ICC_single_	0.45 (0.33–0.58)	0.54 (0.36–0.68)	0.54 (0.36–0.68)
		ICC_average_	0.77 (0.65–0.85)	0.83 (0.67–0.90)	0.83 (0.67–0.90)
	All Weeks	ICC_single_	0.49 (0.39–0.58)	0.48 (0.33–0.59)	0.58 (0.43–0.68)
		ICC_average_	0.80 (0.72–0.85)	0.78 (0.64–0.86)	0.84 (0.74–0.90)
Brow lowering	Week 1	ICC_single_	0.40 (0.29–0.51)	0.32 (0.20–0.43)	0.54 (0.45–0.63)
		ICC_average_	0.72 (0.60–0.81)	0.65 (0.47–0.76)	0.82 (0.76–0.87)
	Week 2	ICC_single_	0.32 (0.23–0.43)	0.21 (0.08–0.34)	0.49 (0.37–0.60)
		ICC_average_	0.66 (0.53–0.75)	0.51 (0.15–0.7)	0.80 (0.69–0.86)
	Week 3	ICC_single_	0.35 (0.22–0.47)	0.18 (0.07–0.30)	0.34 (0.20–0.48)
		ICC_average_	0.68 (0.51–0.79)	0.47 (0.15–0.67)	0.68 (0.45–0.80)
	All Weeks	ICC_single_	0.35 (0.27–0.43)	0.23 (0.13–0.33)	0.46 (0.37–0.55)
		ICC_average_	0.69 (0.59–0.76)	0.54 (0.29–0.69)	0.78 (0.68–0.84)
Lip tightening	Week 1	ICC_single_	0.05 (−0.02 to 0.13)	−0.00 (−0.05 to 0.07)	0.23 (0.14–0.33)
		ICC_average_	0.16 (−0.10 to 0.38)	−0.00 (−0.24 to 0.22)	0.54 (0.38–0.66)
	Week 2	ICC_single_	0.11 (0.03–0.21)	0.04 (−0.02 to 0.10)	0.18 (0.08–0.29)
		ICC_average_	0.33 (0.12–0.51)	0.13 (−0.12 to 0.35)	0.49 (0.18–0.65)
	Week 3	ICC_single_	0.05 (−0.00 to 0.13)	0.03 (−0.03 to 0.10)	0.10 (0.01–0.19)
		ICC_average_	0.18 (−0.10 to 0.40)	0.10 (−0.15 to 0.32)	0.31 (−0.08 to 0.55)
	All Weeks	ICC_single_	0.07 (0.03–0.12)	0.02 (−0.01 to 0.06)	0.17 (0.09–0.25)
		ICC_average_	0.23 (0.05–0.38)	0.09 (−0.08 to 0.24)	0.46 (0.21–0.61)
Hunched posture	Week 1	ICC_single_	0.67 (0.54–0.78)	0.71 (0.57–0.13)	0.83 (0.76–0.88)
		ICC_average_	0.89 (0.82–0.94)	0.91 (0.84–0.95)	0.95 (0.93–0.97)
	Week 2	ICC_single_	0.70 (0.58–0.80)	0.79 (0.69–0.87)	0.69 (0.58–0.79)
		ICC_average_	0.90 (0.85–0.94)	0.94 (0.90–0.96)	0.90 (0.83–0.94)
	Week 3	ICC_single_	0.81 (0.73–0.88)	0.84 (0.77–0.90)	0.70 (0.59–0.79)
		ICC_average_	0.95 (0.92–0.97)	0.96 (0.93–0.97)	0.90 (0.85–0.94)
	All Weeks	ICC_single_	0.74 (0.66–0.80)	0.78 (0.73–0.83)	0.75 (0.69–0.80)
		ICC_average_	0.92 (0.89–0.94)	0.93 (0.91–0.95)	0.92 (0.90–0.94)

Intraclass correlation coefficient (ICC) estimates with respective 95% confidence intervals (95% CI) were calculated using a two–way random effects model for absolute agreement on CGMS scores based on single (ICC_single_) and average (ICC_average_) measures (of 4 raters per cohort, total of 12 raters). The interpretation of ICC values was based ICC_single_ as follows: < 0.5 = poor reliability, 0.5–0.75 = moderate reliability, 0.75–0.9 = good reliability, and > 0.9 = excellent reliability^36^.

Intra-observer reliability of the CMGS scoring was above 0.6 for all raters and ranged from 0.66 (moderate) to 1 (excellent) (Table S7). Male raters had lower intra-observer reliability compared to female raters (p = 0.049).

Cronbach’s alpha coefficient calculated for the overall CMGS scores was 0.81, indicating excellent internal consistency (i.e., only 19% of the final CMGS is due to error variance). Thus, the composite score achieved with the CMGS demonstrates reliability as the individual action units are related to one another. To assess how each individual action unit contributed or changed the reliability of the overall score, the coefficient was recalculated taking each action unit out. The recalculated values were: brow lowering (α_removed_ = 0.78), orbital tightening (α_removed_ = 0.77), lip tightening (α_removed_ = 0.84), and hunched posture (α_removed_ = 0.79). The low variability between the recalculated coefficients and the overall coefficient indicates that all action units contribute similarly to the final score and do not decrease consistency within the tool.

**Figure 3:**
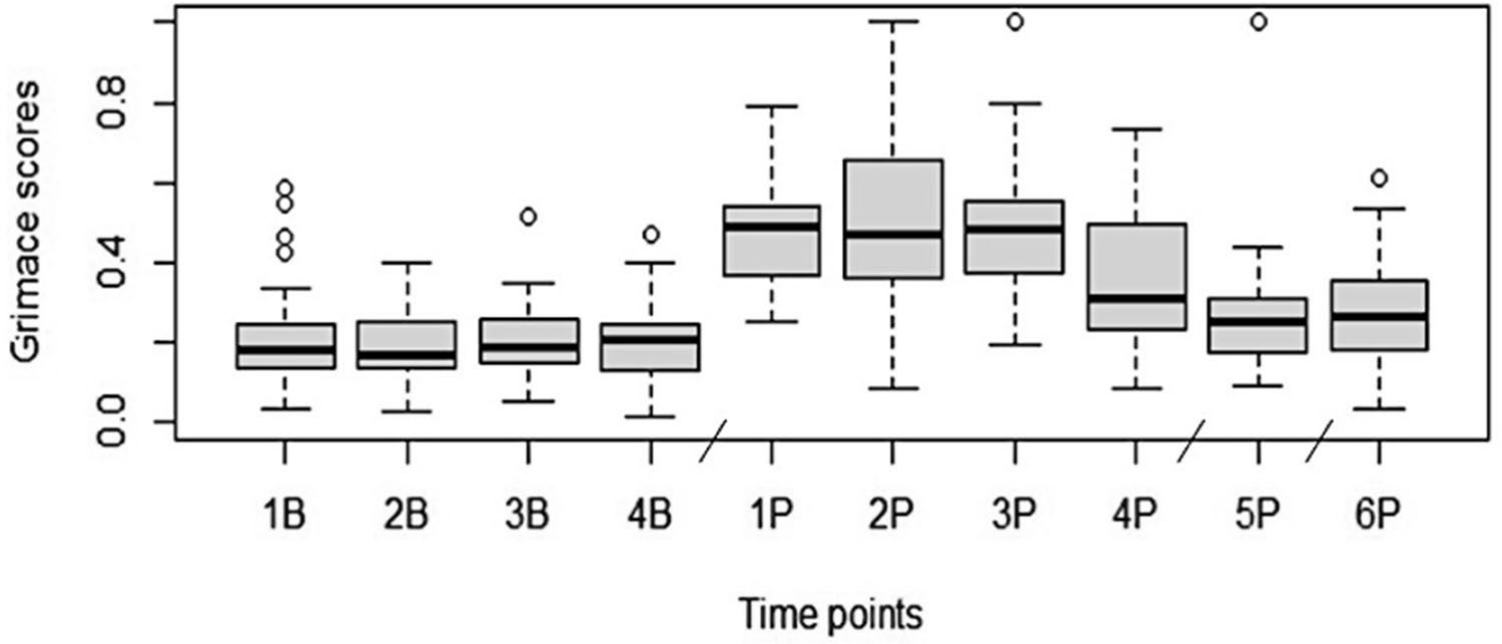
The Cynomolgus Macaque Grimace Scale. Included are descriptions of each action unit, including three facial action units: orbital tightening, brow lowering, and lip tightening, and one postural measure: hunched posture. Each action unit was scored on a 3-point scale based on whether it is not present (score of 0), moderately present (score of 1), or present (score of 2) except lip tightening, which was scored on a 2-point scale based on absence (score of 0) or presence (score of 1).

To assess construct validity, a pairwise comparison with a Tukey’s adjustment was used to compare baseline CMGS scores to the respective post-operative timepoints (see Table S8). There was a significant cohort and sex effect on the CMGS scores (p < 0.05), with male primates and cohort 2 primates (see Table 3) having higher CMGS scores (see Table S8 for LSM of CMGS scores). The four baseline timepoints CMGS score range from 0.19 to 0.21. The first four post-operative timepoints (1P (16:00–17:00: 3 h post-op), 2P (17:00–18:00: 4 h post-op), 3P (18:00–19:00: 5 h post-op), and 4P (approximately 7:00: 17 h post-op) had significantly higher CMGS scores when compared to their respective baseline timepoints (2B (16:00–17:00), 3B (17:00–18:00), 4B (18:00–19:00), and 1B (approximately 7:00) (p < 0.0001) (Table S9). This suggests that breakthrough pain may have occurred during these times. When comparing the two last post-operative time points 5P (17:00–18:00: 24 h post-op), and 6P (approximately 7:00 or 36 h post-op) to their respective baseline timepoints (3B (17:00–18:00), and 1B (approximately 7:00)), no significant differences were found (p > 0.05) (see Table S9), suggesting that breakthrough pain was likely not present for the majority of the primates during those times. CMGS scores are presented as a box plot (Fig. 3).

Of the 12 participants, 11 (92%) reported that the tool would be useful at their research facility. The majority reported that the time to score the 216 images every week required 1–2 h. Subjective confidence levels in using the CMGS varied over time with most participants reporting confidence in using the tool. According to the participants, not all action units were equally easy to score. Nine of 12 participants indicated that lip tightening and three of 12 indicated that brow lowering were the hardest action units to evaluate. Hunched posture, orbital tightening, and brow lowering were the easiest action units to assess.

### Experiment 2: CMGS validation

#### Behavioral observations post-surgery

**Figure 4:**
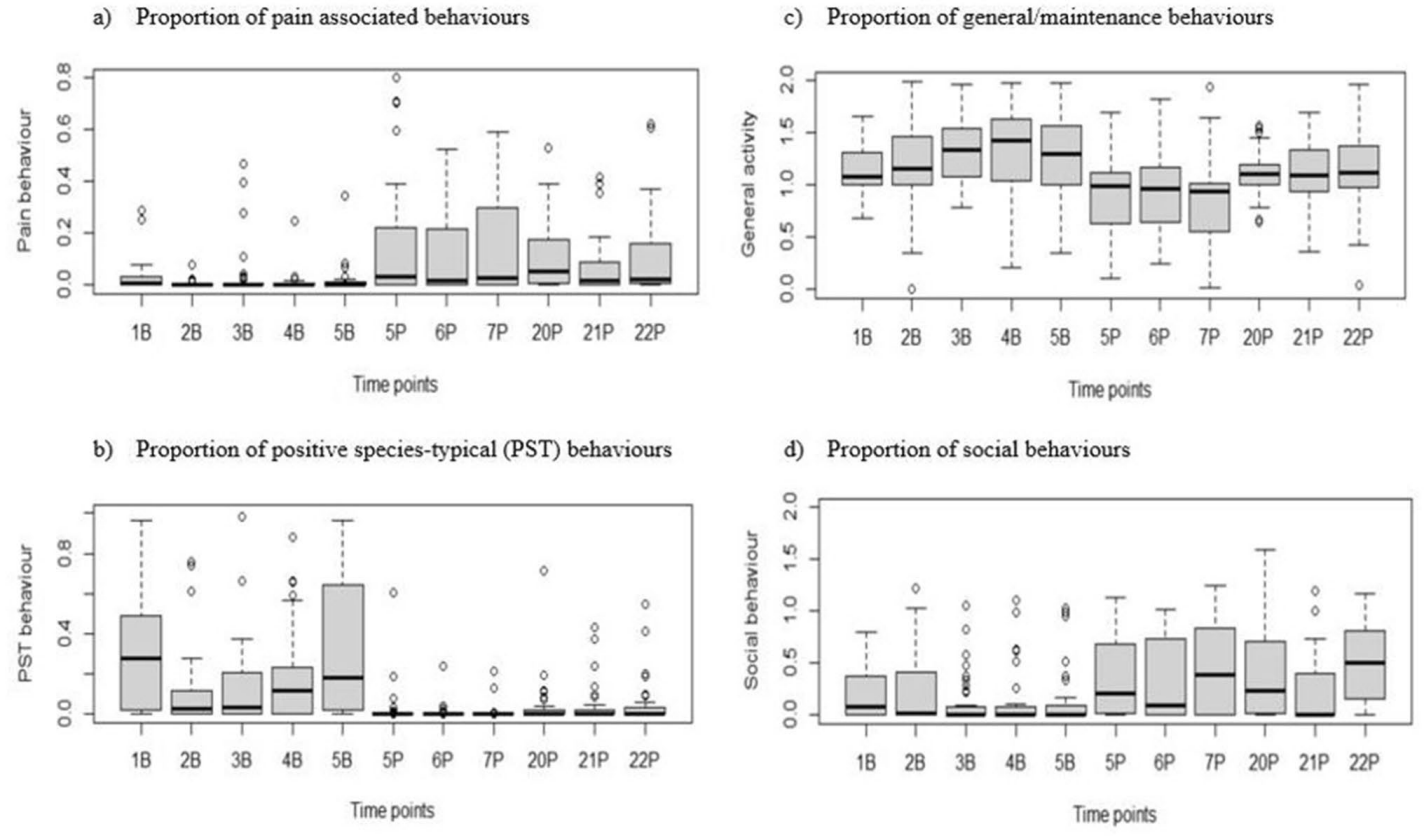
Behavior changes reliably at specific times in primates following surgery related to breakthrough pain. Boxplot showing the proportion of time selected behaviors occur across time from up to 168 h prior to surgery to 24 h post-surgery (n = 43 animals). The solid line indicates the median, the grey box indicates the interquartile range, the whiskers indicate the range, and the circles indicate the extreme values. Timepoints start from up to 168 h before surgery (1B: 06:30–07:30 (cohort 1) or 07:00–08:00 (cohort 2), 2B: 12:00–13:00, 3B:16:00–17:00, 4B:17:00–18:00, 5B:18:00–19:00) and up to 24 h post-surgery ((5P:16:00–17:00 (3 h post-op), 6P:17:00–18:00 (4 h post-op), 7P:18:00–19:00 (5 h post-op), 20P: 06:30–07:30 (cohort 1) or 07:00–08:00 (cohort 2)(17 h post-op), 21P: 12:00–13:00 (20 h post-op), and 22P: 17:00–18:00 (24 h post-op).

There was an effect of sex in that male cynomolgus macaques performed more climbing, self-grooming, inactivity, manipulation of cage resources, and play (p < 0.05). The fixed effect of the interaction between timepoint and sex demonstrated that males performed more self-grooming, spent more time hunched, less time active, and more time embracing in the post-operative period compared to females (p < 0.05). The interaction effect of timepoint per condition and sex demonstrated that males engaged in manipulation of cage resources and play significantly more than females pre- and post-operatively (p < 0.05). There was also an effect of cohort in that primates from cohort 2 performed more foraging and were more inactive (cohort 2: p < 0.05). Primates demonstrated significant behavior changes in the post-operative period (i.e., less general activity and positive species-typical behaviors as well as increased pain-associated behaviors and social behaviors), when compared to the baseline period and lasting up to 5 h post-operative (3P:18:00–19:00, p < 0.05, Table S10). Behaviors within the categories of positive species-typical (manipulation of cage resources), social (embrace/huddle), and pain-associated (self-groom) were significantly different in the post-op period (i.e., less manipulation of cage resources, and increased embracing/huddling and pain-associated behaviors) compared to the baseline period, and lasting up to 24 h post-surgery (5P:17:00–18:00, p < 0.05, Table S10). Behavioral category data was examined using a boxplot across the different timepoints (Fig. 4).

#### Correlation between CMGS and behavior

A significant positive relationship was seen between CMGS scores and pain-associated (rho = 0.35, p < 0.0001), and social behaviors (rho = 0.23, p < 0.0001). There was a significant negative relationship between CMGS scores and general activity (rho = −0.38, p < 0.0001), and positive species-typical behaviors (rho = −0.22, p < 0.0001). Unrelated to pain, a significant positive relationship was noted between human presence in the animal room and primates climbing to a higher level within their enclosure (rho = −0.33, p < 0.0001).

#### Analgesic threshold setting

**Figure 5:**
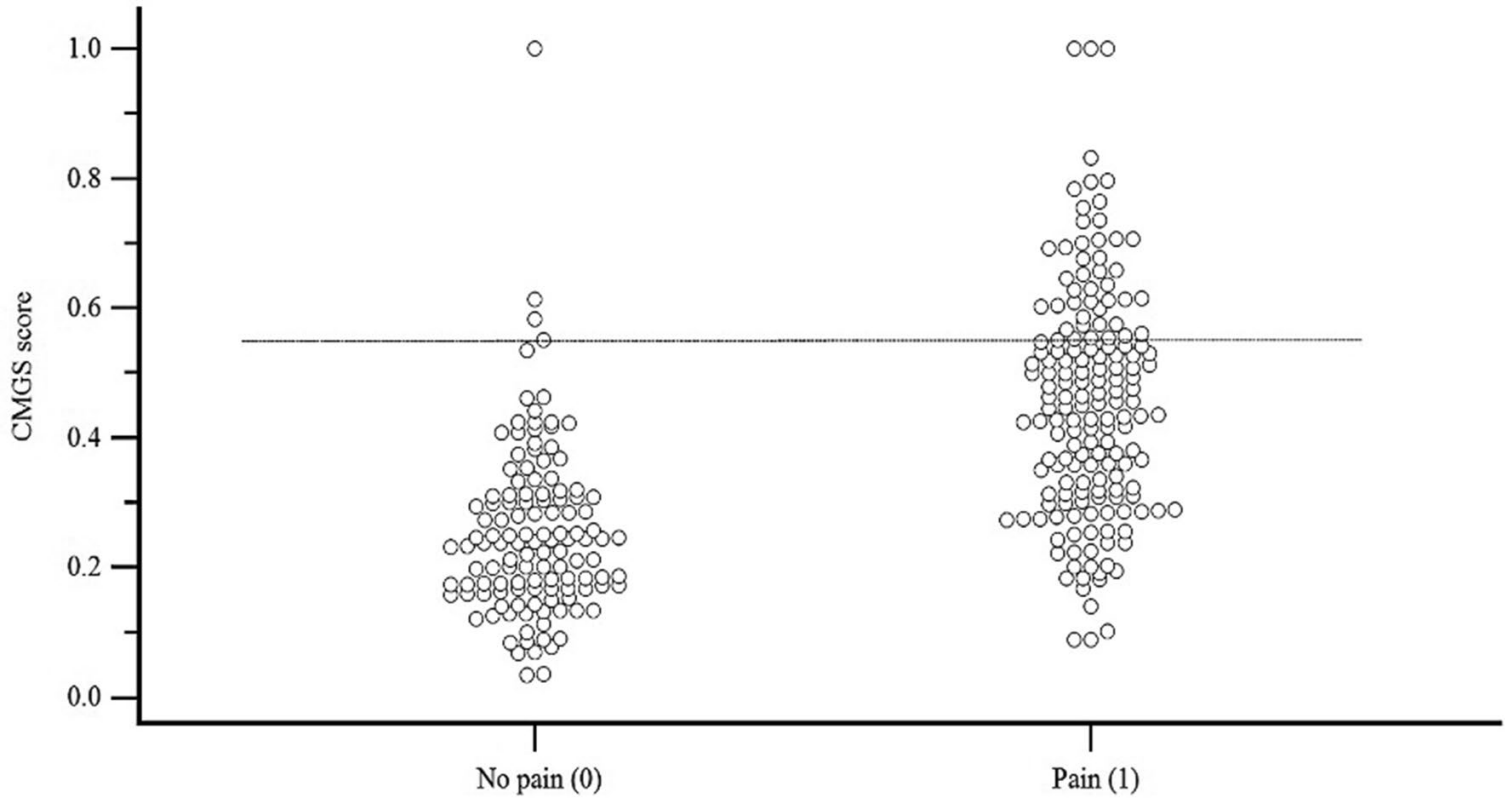
Analgesic threshold curve demonstrating cut-off for rescue pain treatment in cynomolgus macaques. Scatter plot of the 301 CMGS scores categorized as “no pain” (0) or “pain” (1) based on significant behavior changes when pain was detected using post-operative timepoints of 1P, 2P, 3P, and 4P and no pain timepoints (B, 5P, 6P). Images of primate faces and whole-bodies, randomized for timepoint and animal, were also scored by primate veterinary experts based on subjective pain assessment (“no pain” (0) or “pain” (1)) to create an analgesic threshold (0.58), represented by the dashed horizontal line (created with a receiver operating characteristics (ROC) curve). Primates were treated with a multimodal analgesic regimen. Time points start from up to 168 h before surgery for baseline measures (B) and post-operative time points as follows: 1P:16:00–17:00 (3 h post-op), 2P:17:00–18:00 (4 h post-op), 3P:18:00–19:00 (5 h post-op), 4P: 06:30–07:30 (cohort 2) or 07:00–08:00 (cohort 1) (17 h post-op), 5P: 17:00–18:00 (24 h post-op) and 6P:1B: 06:30–07:30 (cohort 2) or 07:00–08:00 (cohort 1) (36 h post-op).

Veterinary experts classified 295 images from seven timepoints pre- and post-surgery. Eight images were discarded as two or more raters noted that they were not possible to score due to image quality. Across all images and timepoints, raters fully agreed on ‘no pain’ for 92 images, agreed on ‘pain for 62 images, and disagreed (one or more raters had different scores for the same image), on 140 occasions. The AUC was determined to be 0.59 (95% CI: 0.55–0.62, p < 0.001). The cut-off score of 0.58 was established based on the value with the best balance between sensitivity (51.7, 95% CI: 47.2–56.1)) and specificity (65.6, 95% CI: 60.6–70.4)). Thus, 0.58 represents the CMGS relative score (equivalent to an absolute score of 4.06) at which a clinical veterinarian or researcher should consider providing or adding an analgesic. Expert CMGS scores are presented in a scatter plot in which timepoints are categorized based on behavior results and compared to the threshold estimate (Fig. 5).

## Discussion

Through the development process of the CMGS four action units related to acute post-surgical pain were identified, with three of the four achieving moderate, good, and excellent reliability, respectively, across observers. The fourth action unit, lip tightening, had poor reliability across observers, which is consistent with some grimace scale features for some other animal species^34^. Once integrated into a formal grimace scale, it was demonstrated that the CMGS could be used to discriminate painful versus non painful macaques and that this was moderately consistent between and within observers. CMGS validity was established as detailed behavioral analysis correlated with CMGS scores, with a positive relationship between CMGS scores pain and social behaviors and a negative relationship between CMGS scores and general activity and positive species-specific behaviors. Finally, an estimated analgesic threshold of 0.58 was determined.

Four parameters consisting of orbital tightening, brow lowering, lip tightening, and hunched posture were compiled for the CGMS. Similar facial action units related to pain have been identified and used in other species. For example, orbital tightening has been described in pain grimace scales in mice^19^, rats^21^ rabbits^27^, ferrets^28^, piglets^23^, and cats^32^ and was recently described as an indicator of pain in Japanese macaques that had undergone a laparotomy^54^. Brow lowering has not been described in the context of pain in any other nonhuman species. A recent study in adult humans evaluating a pain intensity prediction model using facial expressions and electromyography identified several muscles activated when participants reported being in the most pain, including those associated with eyebrow lowering (the corrugator supercilii, depressor supercilii and procerus muscles)^55,56^. Macaques do not have eyebrows, rather they have brow ridges, and a bulging of muscles of the medial brow ridge (i.e., procerus muscle) has been identified as part of their facial actions and correlated to the human muscles responsible for eyebrow lowering and medial contraction^56,57^. Thus, (eye)brow lowering may be a pain-related action unit unique to primates^10,58^. Similarly, lip tightening has not been directly described in other species grimace scales; however, it has been described over 150 years ago by Charles Darwin observing humans experiencing pain [cited by^16^] through to contemporary descriptions^10,16^. More recently, subtle mouth tightening was described post-operatively in Japanese macaques that had undergone a laparotomy^54^. Changes in mouth shape also have been reported for several in grimace scales for other species during pain states, including the horse^13^, cat^14^ lamb^9^, and sheep^10^. Finally, hunched posture or lowered head position in relation to the shoulders is described in other species including cats^14^, sheep^10^, and horses^13^ and although not a facial feature it is important for pain assessment. In a recent study examining wellness indicators of rhesus macaques (*Macaca mulatta*) in the post-operative period, orbital and lip tightening as well as hunched posture were noted to increase in the post-surgical period compared to baseline, similar to our study^26^.

Some action units were seen to be more reliable than others and average ICC values were higher than single ICC values. It is important to report both values^37^, as it informs users about reliability of individual vs multiple scores. In a research setting, multiple observers might assess an animal for pain, such as a caregiver, a veterinarian, and an investigator, increasing reliability. Increased reliability of pain scores for multiple raters has been reported by others using facial grimace scales^18,34,37^. The average ICC values for brow lowering, orbital tightening, and hunched posture demonstrated moderate, good, and excellent reliability, respectively, whereas lip tightening demonstrated poor reliability. This was also noted with the cat, sheep, and horse grimace scales, in which the mouth/lip region was reported to have lower inter- and intra-observer reliability in relation to other areas of the face^22,31,32^. In the current study, participants reported that lip tightening was the hardest action unit to score. This could be because the change in this area is more subtle or ambiguous. For this reason, lip tightening was scored on a 2-point scale (0–1) while a 3-point scale (0–2) was used for other CMGS action units. Another possible explanation for difficulty scoring lip tightening is that observers might have needed knowledge of the individual animal’s baseline facial expression to be able to compare and discriminate changes in lip tightening. Due to the typical process of grimace scale development (i.e., examining blinded pre- and post-operative images), this was not possible and this limitation has been noted by others^16,18^. This emphasizes the importance of daily macaque caregiver observations because of their familiarity with individual animals, including animal personality, appearance, and behavioral characteristics.

Intra-observer reliability of all action units was determined to be moderate to excellent across all participants. However, this study found a gender bias in that males generally had reduced intra-observer reliability. The effect of observer gender has not been studied in the context of grimace scale research but has been studied extensively in human emotion and pain perception research, in which females have been determined to have improved abilities for detecting subtle facial emotions and perceiving pain^59–61^, and whereas males tend to underestimate pain in other people and may rate painful females as being less deserving of support^62,63^. In veterinary medicine, it has been reported that males tend to perceive and assign lower pain scores than females when assessing a potentially painful animal^64^, and this was attributed to reduced sensitivity specifically regarding perception of affective facial expressions^65^. This could explain the lower intra-observer reliability of males and this potential bias should considered when using the tool. More work is needed to further examine potential gender bias related to pain recognition and assessment using the CMGS and other grimace scales. The CMGS demonstrated excellent internal consistency (α = 0.81) and is similar in this regard to other scales including the mouse (α = 0.89)^19^, rat (α = 0.84)^21^, and cat (α = 0.89) grimace scales^32,66^.

The second part of this study assessed the validity of the tool by comparing CMGS scores to periods when pain was presumed to be present (construct validity) and to detailed behavioral analysis (criterion validity). Breakthrough pain was presumed to be present in the first few hours following surgery despite the use of analgesics. In human patients, following a moderately invasive procedure, breakthrough pain is commonly self-reported in patients provided with robust pain treatment from 1 to 8 h after surgery and up to 24 h post-op^46,47^. The results from this study demonstrated a small but significant increase in CMGS scores in the first four post-operative timepoints, with the highest scores occurring 3–5 h post-surgery as well as on the following morning during the first hour of light. This suggests that macaques experienced some pain during these periods. The CMGS scores decreased to almost baseline levels 24 h and 36 h post-surgery. This suggests that pain was well managed and the majority of macaques were not experiencing pain during these periods. Baseline grimace scores in this study were ~ 0.2. As demonstrated with other grimace scales, baseline grimace scores are rarely zero, and it has been postulated that there may be overlap between pain grimacing and other expressions of negative affective state, such as fear, aggression, frustration, digestive discomfort, etc.^18,67,68^. In mice, baseline grimace scores obtained from live and retrospective images from mice of different sex, strains, and ages and baseline grimace scores ranged from 0.2 to 0.3^69^. Knowing context when evaluating facial and postural expressions may assist with reliability when pain scoring. In addition, using the grimace scale in conjunction with behavioral observation of an animal over the course of a minute or two may help to differentiate a fleeting facial expression or postural reaction from more persistent CMGS indicators of a more serious underlying painful condition. Further study is needed with additional animals undergoing different procedures to understand possible confounders for CMGS interpretation.

Pain and response to pain treatment are individual experiences, so inter-individual differences are important to explore. In this study, the average grimace score was highest in the first 4 h of surgery, with a value of 0.49; however, the minimum grimace score was 0.1 and the maximum was 1.0. Given the significant positive correlation between CGMS scores and pain-associated behavior we believe our study to be appropriately powered. Significant interindividual responses to pain have been noted for other primates. For example, in olive baboons (*Papio anubis*) undergoing the same surgery and given the same analgesic treatment (buprenorphine), some individuals demonstrated clinical signs of pain (elevated heart rates) whereas others did not^70^. Pharmacokinetic evidence in macaques administered buprenorphine (0.03 mg/kg) demonstrated high standard deviations of maximum concentration between individuals (Cmax 40.7 ± 48.7 ng/mL) suggesting wide variation in therapeutic effect^71^. This emphasizes the importance of individualized pain recognition, assessment, and treatment and ensuring an appropriate sample size when developing a grimace scale tool.

The behavioral changes observed were well correlated to CMGS scores at the respective timepoints suggesting that the tool has good criterion validity. There was low and moderate correlation between CMGS scores with social behaviors and pain-associated behaviors, respectively. There was also low and moderate inverse correlation between CMGS scores with positive species-typical behaviors and general/maintenance behaviors, respectively. Although these correlations were low to moderate, they are important, especially considering that the macaques in this study were treated with a multimodal analgesic regimen. Despite robust analgesic administration, these significant trends could still be measured. Similar behavior findings been observed in macaques in a post-op wellness study, in which overall activity and arboreal behaviors decreased in the post-operative period and pain-like behaviors such as self-grooming and wound picking increased^26^. In this study, positive species-typical behavior, such as play, exploration, and general activity decreased in the post-operative period. This measure could be comparable to evaluating nest quality in mice to make inferences about animal emotional state. Mice experiencing pain build lower quality nests compared to baseline^72^. The increase in social behavior post-op in macaques in our study highlights the importance of social housing specifically in this period. There is a general lack of research on this subject; however, primates are thought to huddle to thermoregulate more effectively and because it provides them with a sense of comfort and security^73,74^. In veterinary medicine and in research settings, it is common to separate animals for 24 h following surgery because of concerns about infection control and potential overgrooming or overactivity on surgical wounds. Picking at a companion’s wounds was not noted in this study and there were significant positive emotional benefits seen for pairing animals immediately after recovery from anesthesia. In human medicine, patients undergoing the same surgical procedure but with a strong social support network recover faster and require less peri-operative analgesics than patients without social support networks^75,76^. Standard peri-operative practices may need to be re-evaluated in primates to address whether the benefits of social housing immediately after anesthetic recovery outweigh any post-operative risks.

To address the clinical significance of the CMGS an analgesic threshold was established to guide the use of rescue analgesia. The cut-off score for rescue analgesia for the CMGS in the context of this specific surgery was determined to be 0.58. Similar methods were used in this study as for analgesic thresholds developed for cats and rats^32,45^. However, for ethical reasons the macaques evaluated for the threshold in this study were treated with analgesia, thus, the interpretation of the analgesic threshold is slightly different, in that macaques scoring above the threshold would be deemed to require an additional dose of analgesia. In veterinary medicine, as in human medicine, when treating pain many factors need to be considered, such as study restrictions and adverse effects of analgesics, with a goal of achieving a balanced state that allows animals to be comfortable^77^. CMGS users are encouraged to use the threshold to examine how well it performs in practice to ensure macaque comfort.

In this study, male macaques demonstrated higher CMGS scores compared to females as well as other behavioral differences post-surgery. While this might potentially suggest that male macaques experienced more pain or expressed more pain, a sex by condition (pre- vs post-op) interaction was not significant indicating that grimace scores for males were mildly elevated at baseline and post-op. It may be that male macaques inherently have higher grimace scores at rest, due to other affective states, such as aggression. This emphasizes the utility of using the CMGS before and after a painful procedure with specific animals, when possible, to better understand the nature of any changes noted. Sex-related increases in baseline facial grimacing has also been reported in male mice with significantly higher baseline grimace scores reported compared to females^66^. Research on gender differences in relation to pain expression in human medicine are not straightforward with some studies concluding that females have a higher sensitivity to pain, others showing no gender differences, and still others showing that males experienced more pain^78–80^. There is also evidence that pain receptivity and analgesic action mechanisms differ between sexes^81^. One possibility could be that facial expressions of pain in female macaques are more suppressed for fitness purposes^82,83^. Further study is needed to determine other factors modulating baseline grimace scores for macaques and other species.

### Study limitations and future work

There are some limitations to this work and some have already been discussed. For ethical and 3Rs reasons, the study did not have an untreated control group nor an anesthetic-only group. Timepoints during which breakthrough pain might be expected to occur (based on analgesia pharmacokinetics) were chosen to evaluate pain. For this reason, the observed CMGS scores and behavioral correlates may be muted compared to other species grimace scales because macaques were provided with clinically relevant pain control. CMGS scores, behavior findings, and correlations with CMGS scores would be anticipated to be much higher if the macaques had been untreated. Future research could use the CMGS opportunistically when emergency cases occur in a research facility before and after analgesic administration to further evaluate the sensitivity and specificity of the tool. There is also a need to evaluate the CGMS in macaques undergoing anesthesia only. In some but not all strains of mice and rats, grimace scores increase in a limited but consistent manner with administration of inhalant anesthetics, lasting up to 150 min after anesthesia^84–86^. Similarly, some cats undergoing sedation and general anesthesia may experience mildly increased facial grimace scores for up to 30 min following anesthesia^87^. No specific mechanism was proposed for either of these induced grimace score alterations and the presence of hypothermia (a condition commonly occurring during anesthesia and surgery of animals and associated with increased grimace scores in a rat study^88^) was also not reported, so it is unknown whether the changes were induced directly by anesthesia or because of other alterations in physiologic state,. Although the current study did not evaluate macaques until at least 2 h following general anesthesia further research is needed to determine whether and if inhalant or injectable anesthetics have an effect on CMGS scores.

There are several other factors or considerations that are common to grimace scale development across species that should be examined in future research to enhance the CMGS utility. This study evaluated acute pain following one surgical model in sexually immature cynomolgus macaques. While we hypothesize that the CMGS will be generally relevant across different ages of cynomolgus macaques (based on human and animal data demonstrating similarlity of grimace scales across different age groups^13,15,24,25,89^), animals of different ages may experience pain differently and the nature of pain may differ based on the location, duration, and type of tissue damage.Thus, the external validity of the CMGS needs to be assessed for cynomolgus macaques of different ages undergoing different potentially painful procedures. It would also be interesting to evaluate the interspecies utility of the CMGS across other macaque and primate species. Despite the importance of managing and treating pain^4^, there is a lack of evidence of therapeutic analgesic efficacy in primates with dosages often extrapolated from other species^6,7^. Other analgesic regimens should be compared using the CMGS to ensure clinical relevance. It is known that there is an impact of observer training on the reliability and use of grimace scales^18,41,65^. In this study, observer training methods were detailed, but we did not compare between different training methods or on CMGS retention by observers through time and these areas could be further explored. Other factors such as post-operative hypothermia have been demonstrated to alter grimace scores in rats^88^, but were not evaluated in this work. Finally, similar facial action units related to other affective states may occur that overlap with pain-related facial or postural action units. For example, in horses and mice, stressful events and fear can provoke transient facial changes similar to those seen in animals experiencing to pain^18,68,90^. These limitations emphasize the challenges of the grimace scale development, the need to include context and overall behavior in the assessment, and the complexity of factors to consider when evaluating pain.

## Conclusions

In conclusion, this was a two-part study consisting of a proof-of-concept component to develop the CMGS in sexually immature cynomolgus macaques followed by a validation component in which behavioral changes were compared and correlated to CMGS scores. To improve the clinical relevance of the tool an analgesic threshold was established as a cut-off point for administering rescue analgesia. This validated primate pain assessment tool was developed using indirect observation techniques, which are needed to avoid the masking effect of macaque responses to humans and direct observation. Primate pain assessment and management can be challenging and there are many factors that influence facial expressions. Further research could focus on developing an automated system of scoring using the components of the CMGS to avoid human bias and to make the CMGS practical for more routine use.

## Supplementary Information

Supplementary Information.

## Data Availability

All relevant data are within the paper and the supplementary information files.
