# Supplementary material for: Development and validation of a cynomolgus macaque grimace scale for acute pain assessment
Source: Sci Rep. 2023 Feb 24;13:3209. doi: 10.1038/s41598-023-30380-x (PMC9958007; doi:10.1038/s41598-023-30380-x)
Supplement: Supplementary file 1 — Supplementary Information. [file 41598_2023_30380_MOESM1_ESM.docx]

**Supplementary material**

**Table S1**. Subject characteristics, social group, date of surgery, and cohort for all study primates housed in cages (n=44).

| Cage | Animal  ID | Social group | Sex | Age | Surgery date  (dd/mm/yyyy) | Cohort ^L, A^ |
| --- | --- | --- | --- | --- | --- | --- |
| 1 | 1M  2M | 2 | M | 2.12  2.16 | 27/10/2020  27/10/2020 | 1 |
| 2 | 3M  4M | 2 | M | 2.16  2.14 | 28/10/2020  28/10/2020 | 1 |
| 3 | 5M  6M | 2 | M | 2.13  2.16 | 29/10/2020  29/10/2020 | 1 |
| 4 | 7M  8M | 2 | M | 2.13  2.14 | 29/10/2020  29/10/2020 | 1 |
| 5 | 9M  10M  11M | 3 | M | 2.24  2.12  2.15 | 27/10/2020  28/10/2020  30/10/2020 | 1 |
| 6 | 12M  21M  22M | 3 | M | 2.14  2.16  2.25 | 26/01/2021  26/01/2021  26/01/2021 | 2 |
| 7 | 13M  14M | 2 | M | 2.17  2.14 | 02/02/2021  02/02/2021 | 2 |
| 8 | 15M  16M | 2 | M | 2.14  2.13 | 01/01/2021  01/01/2021 | 2 |
| 9 | 17M  18M  19M  20M | 4 | M | 2.18  2.15  2.24  2.19 | 28/01/2021  28/01/2021  28/01/2021  28/01/2021 | 2 |
| 10 | 23F  24F | 2 | F | 2.12  2.15 | *  30/10/2020 | 1 |
| 11 | 25F  26F | 2 | F | 2.12  2.13 | 02/11/2020  02/11/2020 | 1 |
| 12 | 27F  28F | 2 | F | 2.15  2.18 | 03/11/2020  03/11/2020 | 1 |
| 13 | 29F  30F | 2 | F | 2.13  2.12 | 04/11/2020  04/11/2020 | 1 |
| 14 | 31F  32F  33F | 3 | F | 2.12  2.12  2.13 | 02/11/2020  03/11/2020  04/11/2020 | 1 |
| 15 | 34F  43F  44F | 3 | F | 2.16  2.17  2.14 | 25/01/2021  25/01/2021  25/01/2021 | 2 |
| 16 | 35F  36F | 2 | F | 2.13  2.12 | 27/01/2021  27/01/2021 | 2 |
| 17 | 37F  38F | 2 | F | 2.16  2.16 | 27/01/2021  29/01/2021 | 2 |
| 18 | 39F  40F | 2 | F | 2.13  2.14 | 29/01/2021  29/01/2021 | 2 |
| 19 | 41F  42F | 2 | F | 2.14  2.12 | 02/02/2021  01/02/2021 | 2 |

*Does not have a surgery date as the primate died during surgery and was removed from the analysis. The primate was paired with 34F post-surgery.

L: Cohorts differed between 12:12 Dark:Light cycle (±30 min), A: Cohort differed between analgesic protocols (buprenorphine versus slow-release buprenorphine, F: Female, M: Male

**Figure S1**: Detailed summary of image capture and extraction process for CMGS development.


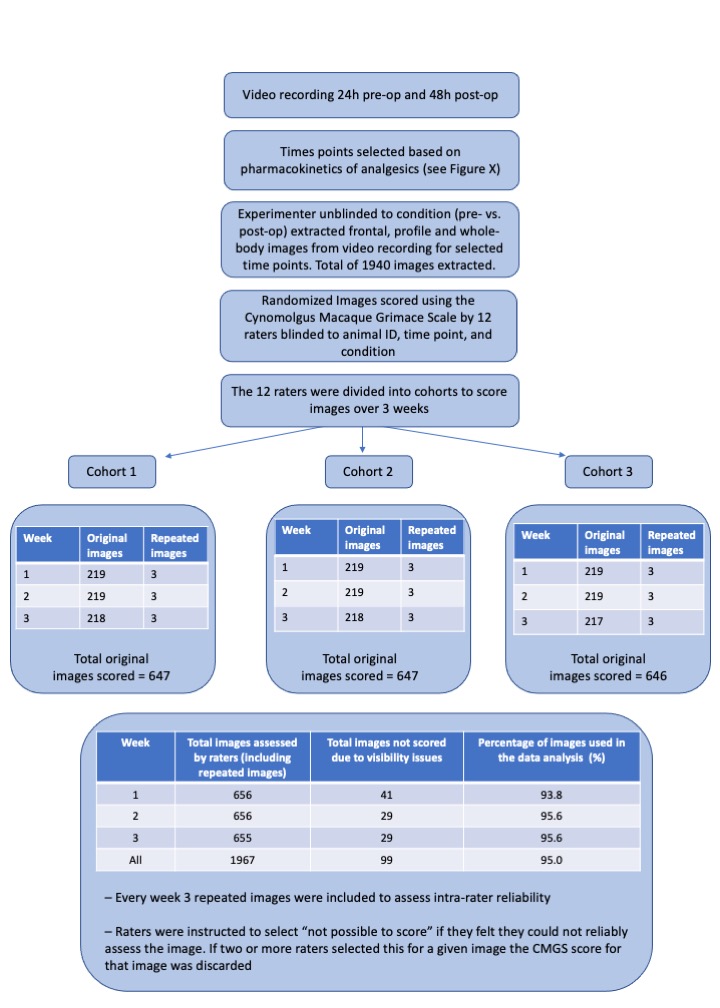


**Table S2.** Number of images captured baseline and post-surgery in cohort 1 males.

| Baseline | Time point | 1M | 2M | 3M | 4M | 5M | 6M | 7M | 8M | 9M | 10M | 11M |
| --- | --- | --- | --- | --- | --- | --- | --- | --- | --- | --- | --- | --- |
|  | 1 | 2F,2P,2WB | 1F,1P,1WB | 1F,1P,1WB | 2F,0P,1WB | 2F,1P,1WB | 3F,1P,1WB | 1F,1P,1WB | 2F,1P,0WB | 2F,1P,1WB | 1F,1P,1WB | - |
|  | 2 | - | - | 0F,1P,1WB | 3F,1P,1WB | - | - | - | 1F,1P,1WB | 2F,1P,1WB | - | - |
|  | 3 | 2F,2P,1WB | 3F,1P,1WB | 1F.1P.1WB | 1F,1P,1WB | 1F,1P,1WB | 3F,1P,1WB | 2F,1P,2WB | 1F,1P,1WB | - | 1F,1P,1WB | 0F,2P,1WB |
|  | 4 | 3F,3P,1WB | 2F,1P,2WB | 1F,1P,1WB | 2F,1P,1WB | 3F,1P,1WB | 1F,1P,1WB | 2F,1P,2WB | 1F,1P,1WB | - | - | 0F,1P,1WB |
| Total  (F,P,WB) |  | 7F,7P,4WB | 6F,3P,4WB | 3F,4P,4WB | 8F,3P,4WB | 6F.3P.3WB | 7F,3P,3WB | 5F,3P,5WB | 5F,4P,3WB | 4F,2P,2WB | 2F,2P,2WB | 0F,3P,2WB |
| Total Baseline |  | 18 | 13 | 11 | 15 | 12 | 13 | 13 | 12 | 8 | 6 | 5 |
| Post-OP | 1 | 2F,3P,1WB | 2F,1P,1WB | 2F,3P,1WB | 1F,1P,1WB | 2F,1P,1WB | 2F,2P,2WB | 1F,1P,1WB | 1F,1P,1WB | - | 1F,1P,1WB | 2F,1P,1WB |
|  | 2 | 3F,3P,2WB | 2F,2P,2WB | 2F,1P,2WB | 2F,1P,1WB | 2F,1P,1WB | 3F,2P,1WB | 2F,1P,1WB | 0F,1P,1WB | 2F,1P,2WB | 2F,1P,2WB | 2F,1P,1WB |
|  | 3 | 2F,4P,2WB | 2F,4P,0WB | 1F,1P,1WB | 2F,1P,1WB | 3F,2P,1WB | 2F,1P,1WB | 1F,2P,1WB | - | 1F,2P,1WB | 2F,2P,2WB | 0F,1P,2WB |
|  | 4 | 0F,1P,1WB | 2F,2P,1WB | 1F,1P,1WB | 1F,1P,1WB | 2F,2P,1WB | 3F,1P,1WB | 1F,1P,1WB | 1F,1P,1WB | 2F,1P,1WB | 2F,1P,2WB | 0F,2P,1WB |
|  | 5 | 1F,3P,2WB | 1F,1P,1WB | 1F,2P,2WB | 1F,1P,1WB | 3F,2P,2WB | 2F,1P,2WB | 1F,2P,2WB | 2F,1P,1WB | 2F,1P,2WB | 1F,2P,2WB | 1F,2P,2WB |
|  | 6 | 2F,2P,2WB | 0F,3P,1WB | 0F,4P,3WB | 1F,1P,1WB | 1F,2P,1WB | 1F,2P,1WB | 1F,1P,1WB | 4F,1P,1WB | 2F,2P,2WB | 1F,2P,1WB | 1F,2P,2WB |
| Total  (F,P,WB) | | 10F,16P,10WB | 9F,13P,6WB | 7F,12P,10WB | 8F,6P,6WB | 13F,10P,7WB | 13F,9P,8WB | 7F,8P,7WB | 8F,5P,5WB | 9F,7P,8WB | 9F,9P,10WB | 6F,9P,9WB |
| Total | | 36 | 28 | 29 | 20 | 30 | 30 | 22 | 18 | 24 | 28 | 24 |
| Over Total | | 54 | 41 | 40 | 35 | 42 | 43 | 35 | 30 | 32 | 34 | 29 |

Cohort 1: Primates underwent surgery in October 2020, had a 12 h light cycle from 07:00 to 19:00

F: Frontal, P: Profile, WB: Whole body

**Table S3.** Number of images captured baseline and post-surgery in cohort 2 males.

| Baseline | Time point | | 12M | 13M | 14M | 15M | 16M | 17M | 18M | 19M | 20M | 21M | 22M |
| --- | --- | --- | --- | --- | --- | --- | --- | --- | --- | --- | --- | --- | --- |
|  | 1 | 0F,1P,0WB | | 2F,2P,2WB | 3F,2P,1WB | 2F,1P,1WB | 0F,1P,2WB | 2F,3P,2WB | 2F,1P,2WB | 0F,2P,1WB | - | 2F,1P,0WB | 1F,1P,1WB |
|  | 2 | 0F,1P,0WB | | 2F,3P,2WB | 2F,2P,2WB | 2F,4P,2WB | 3F,3P,2WB | 2F,3P,3WB | 1F,1P,1WB | 0F,1P,0WB | 1F,1P,1WB | 1F,0P,0WB | 2F,1P,1WB |
|  | 3 | 3F,2P,1WB | | 1F,2P,2WB | 3F,2P,1WB | 1F,3P,1WB | 1F,1P,1WB | 3F,3P,1WB | 2F,2P,2WB | 1F,1P,1WB | 0F,1P,1WB | 2F,0P,0WB | - |
|  | 4 | 3F,1P,3WB | | 2F,1P,2WB | 1F,2P,1WB | 3F,2P,1WB | 0F,1P,1WB | 0F,3P,1WB | 1F,3P,2WB | 0F,1P,1WB | 1F,1P,0WB | 2F,0P,1WB | 0F,3P,1WB |
| Total  (F,P,WB) |  | 6F,5P,4WB | | 7F,8P,8WB | 9F,8P,5WB | 8F,10P,5WB | 4F,6P,6WB | 7F,12P,7WB | 6F,7P,7WB | 1F,5P,3WB | 2F,3P,2WB | 7F,1P,1WB | 3F,5P,3WB |
| Total Baseline |  | 15 | | 23 | 22 | 23 | 16 | 26 | 20 | 9 | 7 | 9 | 11 |
| Post-OP | 1 | 1F,1P,1WB | | 0F,4P,2WB | 4F,2P,2WB | 4F,4P,3WB | 2F,4P,3WB | 2F,3P,4WB | 3F,6P,1WB | 4F,2P,3WB | 8F,1P,2WB | 1F,2P,1WB | 3F,0P,1WB |
|  | 2 | 0F,3P,1WB | | 1F,2P,1WB | 2F,2P,1WB | 3F,4P,2WB | 2F,0P,3WB | 4F,1P,3WB | 3F,1P,2WB | 2F,0P,1WB | 5F,2P,1WB | 4F,2P,2WB | 1F,1P,1WB |
|  | 3 | 1F,2P,1WB | | 2F,2P,2WB | 1F,1P,2WB | 4F,1P,2WB | 1F,0P,1WB | 5F,3P,4WB | 0F,0P,1WB | 4F,0P,1WB | 3F,3P,1WB | 4F,2P,0WB | 2F,0P,0WB |
|  | 4 | 2F,1P,1WB | | 2F,1P,1WB | 1F,2P,3WB | 1F,8P,2WB | 1F,3P,4WB | 6F,2P,2WB | 3F,1P,3WB | 5F,3P,3WB | 1F,2P,1WB | 4F,2P,1WB | 1F,2P,2WB |
|  | 5 | 1F,2P,1WB | | 1F,3P,1WB | 0F,0P,1WB | 1F,2P,1WB | 0F,2P,0WB | 3F,1P,2WB | 0F,0P,1WB | 2F,2P,2WB | 3F,1P,1WB | 3F,2P,3WB | 3F,1P,1WB |
|  | 6 | 1F,2P,1WB | | 1F,3P,2WB | 2F,2P,1WB | 3F,3P,2WB | 2F,1P,3WB | 4F,1P,2WB | 1F,1P,1WB | 1F,1P,1WB | 2F,1P,1WB | - | 1F,2P,1WB |
| Total  (F,P,WB) | | 6F,11P,6WB | | 7F,15P,9WB | 10F,9P,10WB | 16F,22P,12WB | 8F,10P,14WB | 24F,11P,17WB | 10F,9P,9WB | 18F,8P,11WB | 22F,10P,7WB | 16F,10P,7WB | 11F,6P,6WB |
| Total | | 23 | | 31 | 29 | 50 | 32 | 52 | 28 | 37 | 39 | 33 | 23 |
| Over Total | | 38 | | 54 | 51 | 73 | 48 | 78 | 48 | 46 | 46 | 42 | 34 |
| Cohort 2: Primates underwent surgery in January 2021, had a 12 h light cycle from 06:30 to 18:00  F: Frontal, P: Profile, WB: Whole body | | | | | | | | | | | | | |

**Table S4.** Number of images captured baseline and post-surgery in cohort 1 females.

| Baseline | Time  point | 34F | 35F | 36F | 37F | 38F | 39F | 40F | 41F | 42F | 43F | 44F |
| --- | --- | --- | --- | --- | --- | --- | --- | --- | --- | --- | --- | --- |
|  | 1 | 1F,3P,2WB | 4F,1P,1WB | 0F,1P,3WB | 1F,3P,2WB | 3F,1P,2WB | 1F,1P,1WB | 2F,4P,2WB | 1F,1P,3WB | 3F,0P,3WB | 3F,2P,1WB | 1F,1P,1WB |
|  | 2 | 1F,1P,0WB | 2F,1P,1WB | 1F,2P,1WB | 3F,2P,3WB | 4F,1P,2WB | 2F,2P,1WB | 2F,1P,2WB | 1F,1P,1WB | 1F,3P,1WB | 1F,1P,1WB | 0F,1P,1WB |
|  | 3 | 0F,1P,1WB | 2F,1P,1WB | 3F,1P,2WB | 2F,1P,1WB | 2F,2P,1WB | 1F,0P,0WB | 1F,1P,0WB | 1F,1P,2WB | 2F,2P,1WB | 1F,1P,1WB | - |
|  | 4 | 0F,1P,1WB | 2F,3P,1WB | 1F,1P,1WB | 1F,1P,2WB | 2F,0P,1WB | 3F,1P,2WB | 3F,2P,2WB | 4F,1P,1WB | 2F,3P,1WB | 1F,0P,0WB | 0F,2P,1WB |
| Total  (F,P,WB) |  | 2F,6P,4WB | 10F,6P,4WB | 5F,5P,7WB | 7F,7P,8WB | 11F,4P,6WB | 7F,4P,4WB | 8F,8P,6WB | 7F,4P,7WB | 8F,8P,6WB | 6F,4P,3WB | 1F,4P,3WB |
| Total Baseline |  | 12 | 20 | 17 | 22 | 21 | 15 | 22 | 18 | 22 | 13 | 8 |
| Post-OP | 1 | 4F,5P,3WB | 2F,2P,1WB | 2F,1P,1WB | 4F,4P,4WB | 2F,3P,3WB | 2F,4P,2WB | 6F,3P,2WB | 1F,2P,2WB | 2F,4P,5WB | 0F,1P,1WB | 4F,4P,1WB |
|  | 2 | 1F,1P,1WB | 6F,3P,2WB | 4F,1P,1WB | 4F,1P,3WB | 2F,1P,4WB | 3F,4P,3WB | 9F,3P,3WB | 4F,2P,3WB | 0F,4P,2WB | 3F,1P,1WB | 3F,3P,1WB |
|  | 3 | 1F,1P,1WB | 4F,1P,2WB | 1F,1P,2WB | 3F,1P,0WB | 1F,2P,1WB | 0F,3P,2WB | 6F,4P,4WB | 0F,3P,2WB | 4F,1P,3WB | 1F,1P,1WB | 2F,2P,1WB |
|  | 4 | 1F,1P,1WB | 3F,2P,2WB | 3F,3P,3WB | 3F,5P,4WB | 4F,1P,1WB | 1F,2P,1WB | 1F,1P,1WB | 1F,2P,2WB | 3F,2P,4WB | 1F,1P,1WB | 1F,1P,2WB |
|  | 5 | 3F,2P,1WB | 0F,1P,1WB | 1F,0P,1WB | 3F,2P,2WB | 6F,7P,2WB | 2F,0P,1WB | 3F,1P,1WB | 1F,1P,1WB | 2F,3P,2WB | 1F,0P,1WB | 2F,2P,2WB |
|  | 6 | 1F,1P,2WB | 4F,2P,1WB | 2F,1P,1WB | 3F,1P,1WB | 4F,2P,1WB | 2F,5P,2WB | 1F,1P,2WB | 1F,1P,2WB | 2F,3P,2WB | 2F,1P,2WB | 4F,1P,1WB |
| Total | | 11F,11P,9WB | 19F,11P,9WB | 13F,7P,9WB | 20F,14P,14WB | 19F,16P,12WB | 10F,18P,11WB | 26F,13P,13WB | 8F,11P,12WB | 13F,17P,18WB | 8F,5P,7WB | 16F,13P,8WB |
| Total  (F,P,WB) | | 31 | 39 | 29 | 48 | 47 | 39 | 52 | 31 | 48 | 20 | 37 |
| Over Total | | 43 | 59 | 46 | 70 | 68 | 54 | 74 | 49 | 70 | 33 | 45 |

Cohort 1: Primates underwent surgery in October 2020, had a 12 h light cycle from 07:00 to 19:00

F: Frontal, P: Profile, WB: Whole body

**Table S5.** Number of images captured baseline and post-surgery in cohort 2 females.

| Baseline | Time point | 24F | 25F | 26F | 27F | 28F | 29F | 30F | 31F | 32F | 33F |
| --- | --- | --- | --- | --- | --- | --- | --- | --- | --- | --- | --- |
|  | 1 | 2F,1P,1WB | 1F,1P,1WB | 1F,1P,1WB | 2F,1P,1WB | - | 1F,1P,1WB | 1F,1P,2WB | 1F,1P,1WB | 2F,2P,1WB | 2F,2P,1WB |
|  | 2 | - | - | - | 3F,1P,1WB | 1F,1P,1WB | - | 1F,1P,1WB | 0F,1P,1WB | 1F,2P,1WB | 1F,1P,1WB |
|  | 3 | 1F,1P,1WB | - | - | 1F,0P,0WB | 1F,1P,1WB | 1F,1P,1WB | 2F,1P,2WB | 2F,2P,1WB | 1F,1P,1WB | 1F,1P,1WB |
|  | 4 | 2F,1P,1WB | 1F,1P,1WB | - | 2F,1P,1WB | 1F,1P,1WB | 1F,1P,1WB | 1F,1P,2WB | 2F,1P,2WB | 0F,1P,1WB | 1F,2P,2WB |
| Total  (F,P,WB) |  | 5F,3P,3WB | 2F,2P,2B | 1F,1P,1WB | 8F,3P,3WB | 3F,3P,3WB | 3F,3P,3WB | 5F,4P,7WB | 5F,5P,5W | 4F,6P,4WB | 5F,6P,5WB |
| Total Baseline |  | 11 | 6 | 3 | 14 | 9 | 9 | 16 | 15 | 14 | 16 |
| Post-OP | 1 | 1F,1P,1WB | 3F,1P,1WB | 4F,0P,1WB | 1F,1P,1WB | 2F,0P,1WB | 0F,1P,1WB | - | 3F,3P,3WB | 1F,2P,0WB | 0F,2P,2WB |
|  | 2 | 1F,1P,2WB | 2F,1P,1WB | 0F,1P,1WB | 1F,1P,1WB | 0F,1P,1WB | 0F,1P,1WB | - | 3F,2P,2WB | 0F,0P,1WB | 2F,2P,1WB |
|  | 3 | 1F,1P,1WB | 2F,3P,1WB | - | 2F,2P,0WB | 2F,1P,1WB | 0F,1P,1WB | 1F.1P.1WB | 2F,2P,2WB | 1F,0P,1WB | 4F,3P,1WB |
|  | 4 | 2F,1P,1WB | 1F,2P,1WB | 0F,1P,1WB | 1F,2P,1WB | 3F,2P,1WB | 1F,1P,1WB | 1F,1P,1WB | 2F,2P,1WB | 2F,0P,1WB | 0F,4P,1WB |
|  | 5 | 1F.2P.1WB | 2F,3P,1WB | 3F,1P,1WB | 1F,2P,2WB | 2F,2P,2WB | 3F,1P,1WB | 0F,2P,2WB | 1F,2P,2WB | 1F,2P,1WB | 2F,3P,2WB |
|  | 6 | 1F,1P,1WB | 4F,2P,1WB | 1F,1P,2WB | 2F,2P,2WB | 2F,1P,2WB | 0F,3P,1WB | 0F,2P,1WB | 3F,1P,2WB | 1F,2P,2WB | 1F,2P,2WB |
| Total  (F,P,WB) | | 7F,7P,7WB | 14F,12P,6WB | 8F,4P,6WB | 8F,10P,7WB | 11F,7P,8WB | 4F,8P,6WB | 2F,6P,5WB | 14F,12P,12WB | 6F,6P,6WB | 9F,16P,9WB |
| Total | | 21 | 32 | 18 | 25 | 26 | 18 | 13 | 38 | 18 | 34 |
| Over Total | | 32 | 38 | 21 | 39 | 35 | 27 | 29 | 53 | 32 | 50 |

Cohort 2: Primates underwent surgery in January 2021, had a 12 h light cycle from 06:30 to 18:00

F: Frontal, P: Profile, WB: Whole body

Figure S2. Cynomolgus macaque training manual


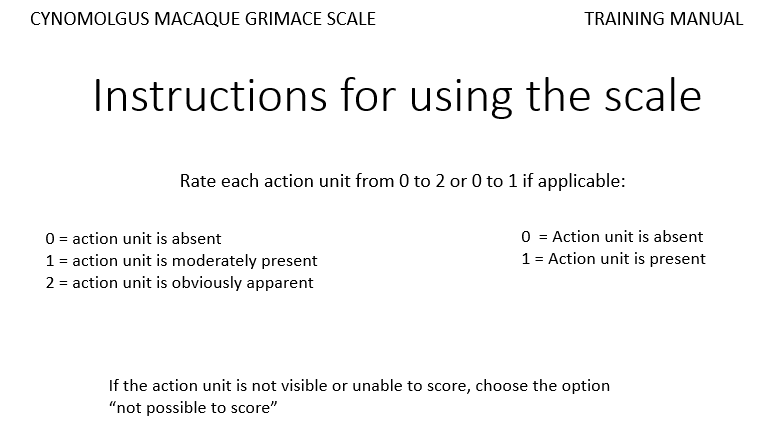


(a)


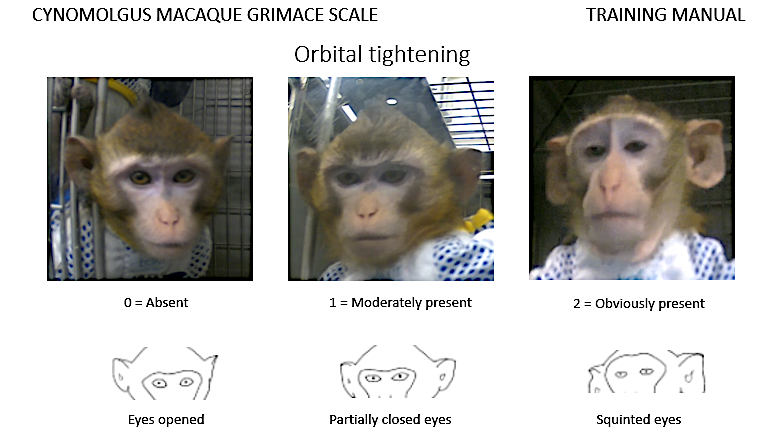


(b)


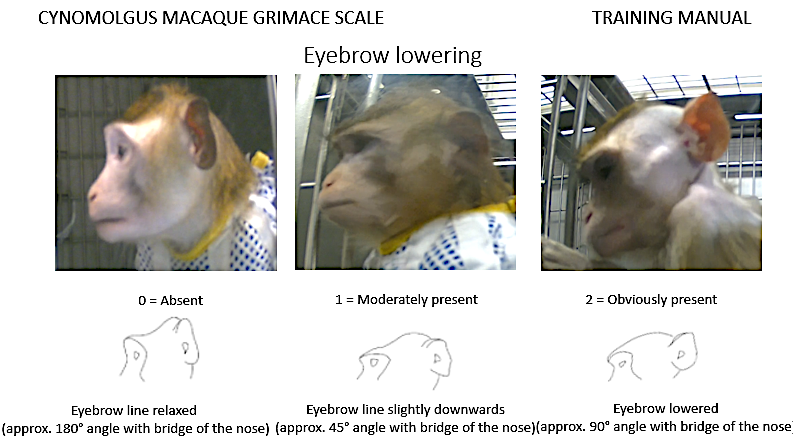


(c)


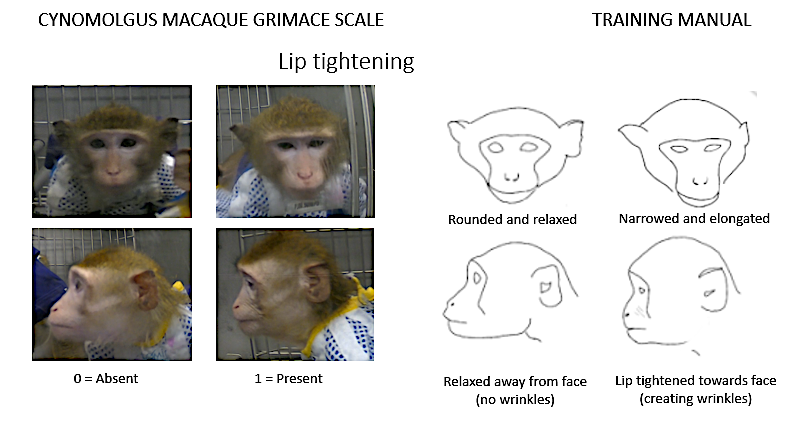


(d)


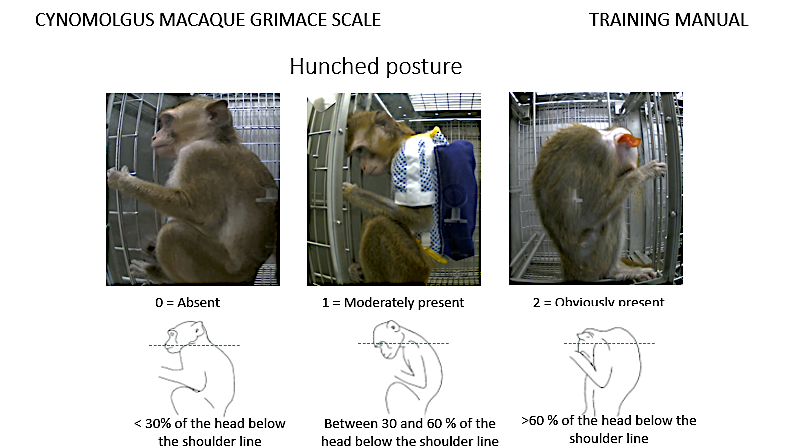


Survey questions

1. Job title
2. Gender
3. How many years have you worked with primates?
4. How much time would you say it took you to score images during Week 1?

Less than 1 h

1 – 2 h

More than 3 h

1. How much time would you say it took you to score images during Week 2?

Less than 1 h

1 – 2 h

More than 3 h

1. How much time would you say it took you to score images during Week 3?

Less than 1 h

1 – 2 h

More than 3 h

1. How confident did you feel when scoring images Week 1 on a scale from 0 -2?

0: Not confident

1: Confident

2: Very confident

1. How confident did you feel when scoring images Week 2 on a scale from 0 -2?

0: Not confident

1: Confident

2: Very confident

1. How confident did you feel when scoring images Week 3 on a scale from 0 -2?

0: Not confident

1: Confident

2: Very confident

1. Do you think this is a useful tool that you could use at your facility?

Yes

No

1. Do you think it would be straight forward to train technical staff to use this tool?

Yes

No

1. In your opinion which was the most difficult parameter to score?

Eyebrow lowering

Lip tightening

Orbital tightening

Posture

1. In your opinion which was the easiest parameter to score?

Eyebrow lowering

Lip tightening

Orbital tightening

Posture

1. Please write out any other comments about your experience using the grimace scale?

**Table S6.** Sampling strategy of 6 subject characteristics and time point assessed.

| Cage | Animals  ID | Social group | Sex | Age | Time of day and condition |
| --- | --- | --- | --- | --- | --- |
| 7 | 13M  14M | 2 | M | 2.17  2.14 | 19:00-20:00  (baseline) |
| 8 | 15M  16M | 2 | M | 2.14  2.13 | 17:00 -18:00  (post-op) |
| 16 | 35F  36F | 2 | F | 2.13  2.12 | 16:00-17:00  (baseline) |

F: Female, M: Male

Table S7. Intra-observer reliability of 12 raters of the cynomolgus macaque grimace scale.

| Cohort | 1 | | | | 2 | | | | 3 | | | |
| --- | --- | --- | --- | --- | --- | --- | --- | --- | --- | --- | --- | --- |
| Rater | 3 | 6 | 10 | 12 | 5 | 7 | 8 | 9 | 1 | 2 | 4 | 11 |
| Gender | F | M | F | M | F | M | F | M | F | F | F | M |
| ICCsingle (95% CI) | 0.94 (0.85-0.97) | 0.71 (0.34-0.87) | 0.62 (0.17-0.85) | 0.67 (0.33-0.86) | 0.79 (0.58-0.91) | 0.66 (0.35-0.84) | 0.86 (0.71-0.94) | 0.68 (0.34- 0.85) | 0.85 (0.60-0.95) | 1  (-) | 1  (-) | 0.66 (0.25-0.87) |

Intraclass correlation coefficient (ICC) estimates with respective 95% confidence intervals (95% CI) were calculated using a two-way random effects model for absolute agreement based on single (ICC_single_) and average (ICC_average_) measures (4 raters per cohort, total of 12 raters). The interpretation of ICC values was based ICC_single_ and is as follows: below 0.5 = poor reliability, between 0.5 and 0.75 = moderate reliability, between 0.75 and 0.9 = good reliability, and above 0.9 = excellent reliability (Koo and Li, 2016).

Table S8. Facial grimace scores in relation to pre- and post-surgical time points (N=43 primates).

|  | Time  points | Absolute time | Time relative to surgery (h) | LS Mean  (95% CI) | Standard  Error |
| --- | --- | --- | --- | --- | --- |
| Baseline | 1B | ^1^6:30-7:30  ^2^7:00-8:00 | -72 - 168 | 0.21 (0.14-0.28) | 0.02 |
|  | 2B | 16:00-17:00 | -72 -168 | 0.19 (0.11-0.26) | 0.02 |
|  | 3B | 17:00-18:00 | -72 -168 | 0.20 (0.13-0.27) | 0.02 |
|  | 4B | 18:00-19:00 | -72 -168 | 0.19 (0.13-0.27) | 0.02 |
| Post-Op | 1P | 16:00-17:00 | +3 | 0.47 (0.39-0.54) | 0.02 |
|  | 2P | 17:00-18:00 | +4 | 0.49 (0.42-0.56) | 0.02 |
|  | 3P | 18:00-19:00 | +5 | 0.48 (0.41-0.54) | 0.02 |
|  | 4P | ^1^6:30-7:30  ^2^7:00-8:00 | +17 | 0.36 (0.29-0.42) | 0.02 |
|  | 5P | 17:00-18:00 | +24 | 0.27 (0.20-0.33) | 0.02 |
|  | 6P | ^1^6:30-7:30  ^2^7:00-8:00 | +36 | 0.28 (0.21-0.35) | 0.02 |

Least-square (LS) means and their 95% confidence interval (95% CI) were calculated using a Gaussian linear mixed model (n=43) and respective standard errors are presented.

^-^Indicative of pre-surgical period

^+^Indicative of post-surgical period

^1^Cohort 1 animals (different time due to different light cycle)

^2^Cohort 2 animals (different time due to different light cycle)

^B^Baseline period

^P^Post-operative period

Table S9. Pre- and post-operative Cynomolgus Macaque Grimace Scale (CMGS) score respective time interval comparison.

| **Time point comparison** | **Absolute time comparison** | | **Size effect estimate (±SE) (P value)** |
| --- | --- | --- | --- |
| 1P-2B | | 16:00-17:00 | 0.28 (0.03) **(p<0.0001)** |
| 2P-3B | | 17:00-18:00 | 0.29 (0.03) **(p<0.0001)** |
| 3P-4B | | 18:00-19:00 | 0.28 (0.03) **(p<0.0001)** |
| 4P-1B | | ^1^6:30-7:30  ^2^7:00-8:00 | 0.15 (0.03) **(p<0.0001)** |
| 5P-3B | | 17:00-18:00 | 0.07 (0.03) (p=0.208) |
| 6P-1B | | ^1^6:30-7:30  ^2^7:00-8:00 | 0.07 (0.03) (p=0.188) |

Absolute size effect estimates, and their standard error (SE) and P values were calculated using a pairwise comparison with a Turkey’s adjustment for multiple comparison. Significant effects are indicated in bold.

^1^Cohort 1 animals (different time due to different light cycle)

^2^Cohort 2 animals (different time due to different light cycle)

^B^Baseline period

^P^Post-operative period

Table S10. Comparison of pre- and post-operative behaviour effect size estimates (mean ± SE) of respective time interval comparison (absolute values presented).

|  | | Behaviour Category | | | | | | | | | |
| --- | --- | --- | --- | --- | --- | --- | --- | --- | --- | --- | --- |
|  |  | Positive species-typical | | | General activity/maintenance | | Social interactions | | Pain-associated behaviours | | |
| Time point comparison | Absolute time comparison | Forage | Play | MCR* | Active | Eat | Allo-grooming | Embrace/  huddle | MVTW* | Hunched | Self-groom |
| 5P-3B | 16:00-17:00 | 0.06  (0.03)  p=0.8041 | 0.02  (0.03)  p=1.0000 | 0.10  (0.04)  p=0.1708 | 0.23  (0.04)  **p<0.0001** | 0.25  (0.07)  **p=0.0098** | 0.00  (0.01)  p=1.0000 | 0.26  (0.07)  **p=0.0130** | 0.04  (0.02)  p=0.5520 | 0.17  (0.04)  **p=0.0034** | 0.01 (0.02)  p=1.0000 |
| 6P-4B | 17:00-18:00 | 0.16  (0.03)  **p=0.0002** | 0.08  (0.03)  p=0.3542 | 0.17  (0.04)  **p=0.0005** | 0.17  (0.04)  **p=0.0014** | 0.28  (0.07) **p=0.0013** | 0.00  (0.01)  p=1.000 | 0.23  (0.07)  p=0.0502 | 0.06  (0.02)  p=0.0683 | 0.17  (0.04) **p=0.0023** | 0.01  (0.02)  p=1.0000 |
| 7P-5B | 18:00-19:00 | 0.16  (0.03)  **p=0.0001** | 0.12  (0.03)  **p=0.0174** | 0.24  (0.04)  **p<0.0001** | 0.24  (0.04)  **p<0.0001** | 0.28  (0.06)  **p=0.0010** | 0.00  (0.01)  p=1.0000 | 0.31  (0.07)  **p=0.0004** | 0.07  (0.02)  **p=0.0391** | 0.20  (0.04)  **p<0.0001** | 0.03  (0.02)  p=0.9765 |
| 20P-1B | ^1^6:30-7:30  ^2^7:00-8:00 | 0.09  (0.03)  p=0.2530 | 0.23  (0.03)  **p<0.0001** | 0.14  (0.04)  **p=0.0057** | 0.00  (0.04)  p=1.0000 | 0.02  (0.06)  p=1.0000 | 0.06  (0.01)  **p=0.0003** | 0.10  (0.07)  p=0.9577 | 0.11  (0.02)  **p<0.0001** | 0.02  (0.04)  p=1.0000 | 0.07  (0.02)  p=0.0510 |
| 21P-2B | 12:00-13:00 | 0.05  (0.04)  p=0.9780 | -0.02  (0.04)  p=1.0000 | 0.08  (0.04)  p=0.6437 | 0.02  (0.04)  p=1.0000 | 0.02  (0.07)  p=1.0000 | 0.02  (0.01) p=0.9848 | 0.01  (0.07)  p=1.0000 | 0.06  (0.02)  p=0.2311 | 0.08  (0.04)  p=0.7879 | 0.06  (0.02)  p=0.2196 |
| 22P- 4B | 17:00-18:00 | 0.10  (0.03)  p=0.1274 | 0.03  (0.03)  p=0.9989 | 0.16  (0.04)  **p=0.0012** | 0.07  (0.04)  p=0.7864 | 0.15  (0.07)  p=0.4828 | 0.02  (0.01) p=0.8482 | 0.36  (0.07)  **p<0.0001** | 0.05  (0.02)  p=0.2910 | 0.11  (0.04)  p=0.3135 | 0.08  (0.02)  **p=0.0201** |

Absolute size effect estimates, and their standard error (SE) and P values were calculated using a pairwise comparison with a Turkey’s adjustment for multiple comparison. Significant effects are indicated in bold.

*MVTW: Movement Directed Towards the Wound; MCR: Manipulation of Cage Resources

^1^Cohort 1 animals (different time due to different light cycle)

^2^Cohort 2 animals (different time due to different light cycle)

^B^Baseline period

^P^Post-operative period
